# Cell–cell fusion induced by reovirus FAST proteins enhances replication and pathogenicity of non-enveloped dsRNA viruses

**DOI:** 10.1371/journal.ppat.1007675

**Published:** 2019-04-25

**Authors:** Yuta Kanai, Takahiro Kawagishi, Yusuke Sakai, Ryotaro Nouda, Masayuki Shimojima, Masayuki Saijo, Yoshiharu Matsuura, Takeshi Kobayashi

**Affiliations:** 1 Department of Virology, Research Institute for Microbial Diseases, Osaka University, Osaka, Japan; 2 Laboratory of Veterinary Pathology, Joint Faculty of Veterinary Medicine, Yamaguchi University, Yamaguchi, Japan; 3 Department of Virology I, National Institute of Infectious Diseases, Tokyo, Japan; 4 Department of Molecular Virology, Research Institute for Microbial Diseases, Osaka University, Osaka, Japan; Indiana University, UNITED STATES

## Abstract

Fusogenic reoviruses encode fusion-associated small transmembrane (FAST) protein, which induces cell–cell fusion. FAST protein is the only known fusogenic protein in non-enveloped viruses, and its role in virus replication is not yet known. We generated replication-competent, FAST protein-deficient pteropine orthoreovirus and demonstrated that FAST protein was not essential for viral replication, but enhanced viral replication in the early phase of infection. Addition of recombinant FAST protein enhanced replication of FAST-deficient virus and other non-fusogenic viruses in a fusion-dependent and FAST-species-independent manner. In a mouse model, replication and pathogenicity of FAST-deficient virus were severely impaired relative to wild-type virus, indicating that FAST protein is a major determinant of the high pathogenicity of fusogenic reovirus. FAST-deficient virus also conferred effective protection against challenge with lethal homologous virus strains in mice. Our results demonstrate a novel role of a viral fusogenic protein and the existence of a cell–cell fusion-dependent replication system in non-enveloped viruses.

## Author summary

Among diverse viral proteins of non-enveloped viruses, only FAST protein encoded by fusogenic reoviruses belonging to the family *Reoviridae* induces cell–cell fusion during viral replication cycle. Unlike enveloped viruses, non-enveloped viruses do not require fusion proteins to enter cells. Although the biochemical characteristics of FAST protein have been extensively studied, its biological function and its role in viral replication remain unknown. Here, we showed that cell–cell fusion induced by FAST protein dramatically increased replication of non-enveloped dsRNA viruses that did not encode FAST protein. We also demonstrated that FAST mutant viruses could be used to generate live viral vaccines. This study reports the unprecedented finding that a viral non-structural protein enhances replication of non-enveloped dsRNA viruses by inducing cell–cell fusion.

## Introduction

Proteins of the fusion-associated small transmembrane (FAST) family, which are encoded by some members of the *Reoviridae* family, are the only viral fusogenic proteins known in non-enveloped viruses, which do not require fusion to enter the host cell [1]. FAST proteins are small (95–198 amino acids) and are expressed as non-structural proteins during the viral replication cycle [2]. FAST proteins induce syncytium formation by fusion of host cells, such as epithelial cells and fibroblasts [1,3,4]. By contrast, fusogenic peptides and proteins of enveloped viruses are essential components of virion structure that are required for fusion between the viral membrane (envelope) and the cellular membrane, which is required for viral entry into the cell.

The *Reoviridae* family is composed of 15 genera, including rotaviruses and orthoreoviruses, both of which include common human pathogens. Among the members of the *Reoviridae* family, several types of FAST protein are known. In the genus *Orthoreovirus*, avian orthoreovirus (ARV) and pteropine orthoreovirus (PRV) encode FAST-p10, whereas baboon orthoreovirus (BRV), Broome virus, and reptilian orthoreovirus (RRV) encode FAST-p15, FAST-p13, and FAST-p14, respectively [1,3–5]. In the genus *Aquareovirus*, FAST-p22 is encoded by group A aquareovirus, and FAST-p16 is encoded by group C and G aquareoviruses [6–8]. Those fusogenic reoviruses coding FAST proteins have important implications for public health and the poultry industry. A common human pathogen, mammalian orthoreovirus (MRV), which causes asymptomatic infection in respiratory and intestinal organs [9], is the only known non-fusogenic virus in the genus *Orthoreovirus*.

FAST proteins are composed of an N-terminal ectodomain, a transmembrane domain, and a C-terminal cytoplasmic domain [10]. The results of mutagenesis of recombinant FAST proteins indicate that each domain is required for fusion activity [3,11–18]. Because of their small size and simple structure, the non-structural FAST proteins are not thought to be related to the structural enveloped virus fusion proteins [1]. Viral fusion proteins are structurally divided into four classes: class I, with a characteristic α-helix trimer (as in human immunodeficiency virus (HIV) gp41); class II, with a β-sheet-based elongated ectodomain (as in dengue virus glycoprotein); class III, composed of an α-helix and β-sheet combined ectodomain (as in rabies virus G glycoprotein); and class IV, which is the FAST protein family [19].

ARV is a common avian pathogen that has been studied extensively as a prototype of fusogenic reoviruses, and syncytium formation is known to occur after infection by ARV [20]. Results with natural variants of ARV demonstrated that the level of fusion activity is associated with the degree of pathogenicity in chicken embryos, but does not affect viral replication *in vitro* [21]. The use of protein-transport inhibitors (including brefeldin A and tunicamycin) reduces syncytium formation in ARV-infected cells, and inhibits but does not prevent egress of synthesized virion [22]. Recombinant vesicular stomatitis virus (VSV) expressing RRV FAST-p14 has unaltered viral replication *in vitro*, but enhanced replication in mouse brains, with significant increase of pathogenicity compared with VSV expressing green fluorescent protein [23]. Further evidence of the association between FAST proteins and viral pathogenicity has come from epidemiological and clinical studies of ARV in chickens with arthritis [24], BRV in baboons with encephalitis [25], and RRV in snakes with interstitial pneumonia [26]. Addition of FAST protein dramatically enhances production of non-fusogenic MRV and group A rotavirus (RVA), enabling the improvement of plasmid-based reverse genetics systems for these viruses [27]. Even with the results from these studies, no direct evidence has previously been produced for the association of cell–cell fusion by FAST with viral replication and spread.

The lack of reverse genetics systems for fusogenic reoviruses has been the main obstacle to study of the biological functions of FAST proteins. We have developed a plasmid-based reverse genetics system for PRV, a fusogenic bat-borne reovirus [28]. PRV has a double-stranded (ds)RNA genome with 10 segments and was isolated from fruit bats in Nelson Bay, Australia, in 1968 [29]. In the past decade, PRV was isolated from patients with high fever, sore throat, cough, and/or diarrhea in south-eastern Asian countries, in addition to detection in bats [30], suggesting that PRV is a bat-borne zoonotic virus.

In this study, we examined the properties of various mutant PRV strains and recombinant FAST proteins. The results demonstrated that cell–cell fusion mediated by FAST protein increases viral replication. In a lethal mouse-infection model, FAST protein was the critical determinant of viral pathogenesis. Introduction of amino acid substitutions in PRV FAST protein produced an attenuated vaccine strain that completely protected animals from lethal infection.

## Results

### Generation of FAST protein-deficient viruses

**Fig 1:**
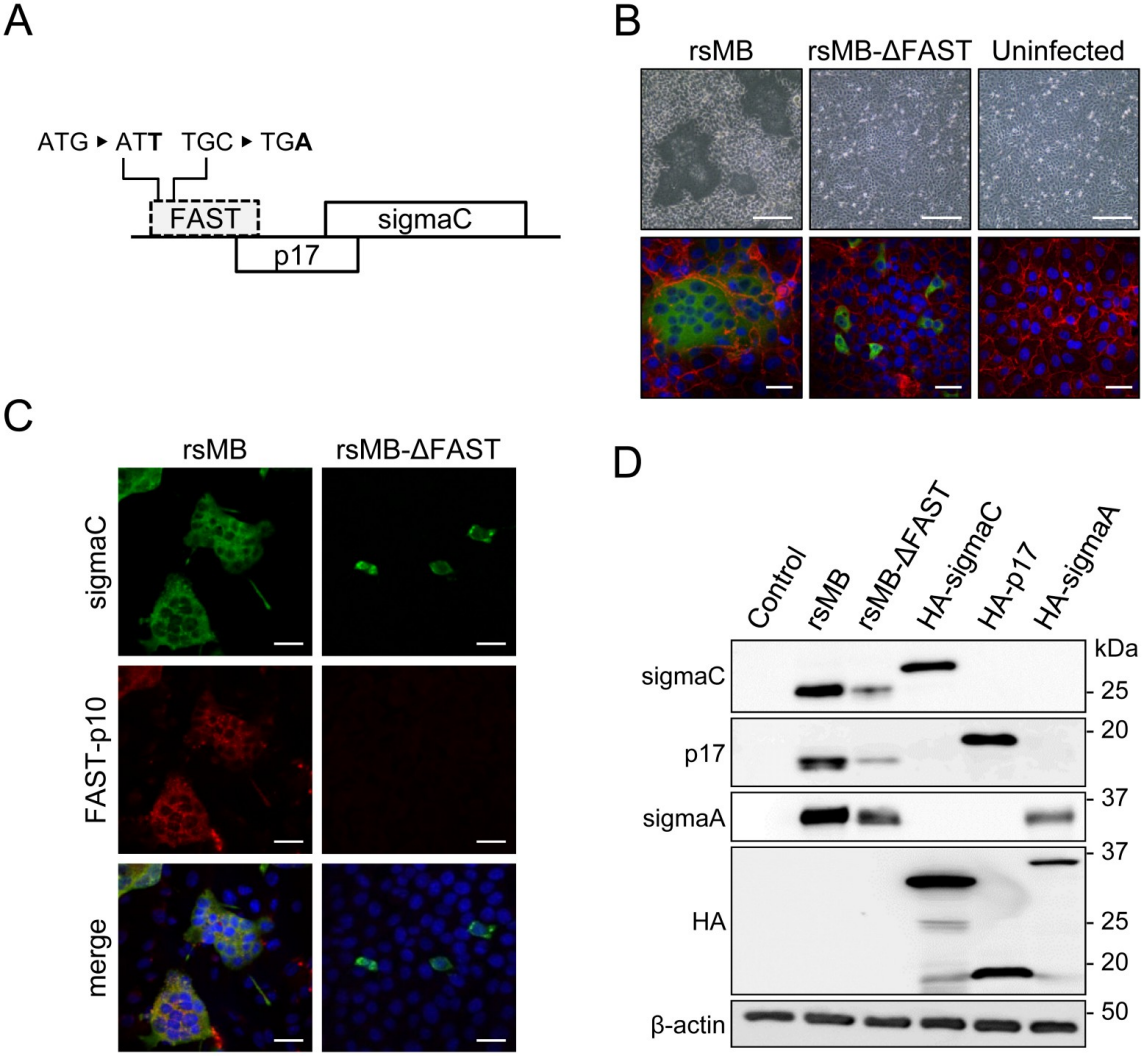
Generation of FAST protein-deficient recombinant pteropine orthoreovirus (PRV). (*A*) Construction of FAST protein-deficient PRV strain MB (rsMB-ΔFAST) involved generation of S1 gene segment with replacement of the first codon of the FAST-p10 ORF (ATG) with ATT and the fifth codon (TGC) with TGA. (*B*, *C*) Syncytia are formed by wild-type rsMB, but not rsMB-ΔFAST. (*B*) Vero cells infected with rsMB or rsMB-ΔFAST were fixed, and viral antigen (sigmaC) was detected by indirect immunofluorescence assay with rabbit anti-sigmaC antibody followed by a CF488-conjugated secondary antibody. Cellular plasma membranes were visualized with anti-pan-cadherin antibody and CF594-conjugated secondary antibody (red). Phase contrast images (top) and fluorescence images (bottom) are shown. Scale bars are 200 μm (top) and 20 μm (bottom). (*C*) BSR cells infected with rsMB or rsMB-ΔFAST were fixed at 16 h post infection. Viral sigmaC (green) and FAST-p10 (red) antigens were visualized by indirect immunofluorescence assay with antibodies to sigmaC and FAST-p10 (residues 21–40), respectively. Scale bars are 200 μm. (*D*) Structural and non-structural viral protein expression in cells infected with rsMB or rsMB-ΔFAST. Whole-cell lysates of Vero cells infected with rsMB or rsMB-ΔFAST at a MOI of 0.1 PFU/cell and harvested at 24 h post infection were subjected to western blotting. Viral proteins were detected with anti-sigmaC, anti-p17, and anti-sigmaA antibodies. Plasmid vectors directing expression of hemagglutinin (HA)-sigmaC, HA-p17, and HA-sigmaA were transfected as positive controls for antibody detection, and β-actin staining was a control for protein loading.

We used a reverse genetics system to generate recombinant PRV strain Miyazaki-Bali/2007 with (rsMB) or without (rsMB-ΔFAST) FAST-p10 expression [28]. The rsMB-ΔFAST virus was recovered from cells transfected with rescue plasmids of nine intact PRV-MB gene segments and one segment (S1) with disruption of the FAST-p10 translational start codon and insertion of a stop codon (Fig 1A). Syncytium formation was detected in cells infected with rsMB, but not in those infected with rsMB-ΔFAST (Fig 1B). Staining of the viral attachment protein sigmaC identified infection of individual cells by rsMB-ΔFAST (Fig 1B). Immunofluorescence with FAST-p10-specific antiserum detected FAST-p10 protein in cells infected with rsMB, but not in those infected with rsMB-ΔFAST (Fig 1C). To confirm this result, we also generated another FAST-p10-deficient virus (rsMB-ΔFAST181) by deletion of S1 nucleotides 85–265, corresponding to the FAST-p10 open reading frame (ORF) (S1A Fig). Electropherotype analysis confirmed this deletion (S1B Fig). Although sigmaC was detected in individual cells following infection with rsMB-ΔFAST181, syncytium formation was not detected (S1C Fig).

The PRV S1 gene segment contains three ORFs, encoding FAST-p10, p17, and sigmaC. Atypical ribosome shunting and leaky scanning have been speculated to underlie S1 tri-cistronic expression [31], suggesting that deficiency of the FAST-p10 ORF might disturb expression of p17 and sigmaC. However, immunoblotting demonstrated that p17 and sigmaC were both expressed in cells infected with rsMB-ΔFAST (Fig 1D).

### Replication of FAST protein-deficient virus in cultured cells

**Fig 2:**
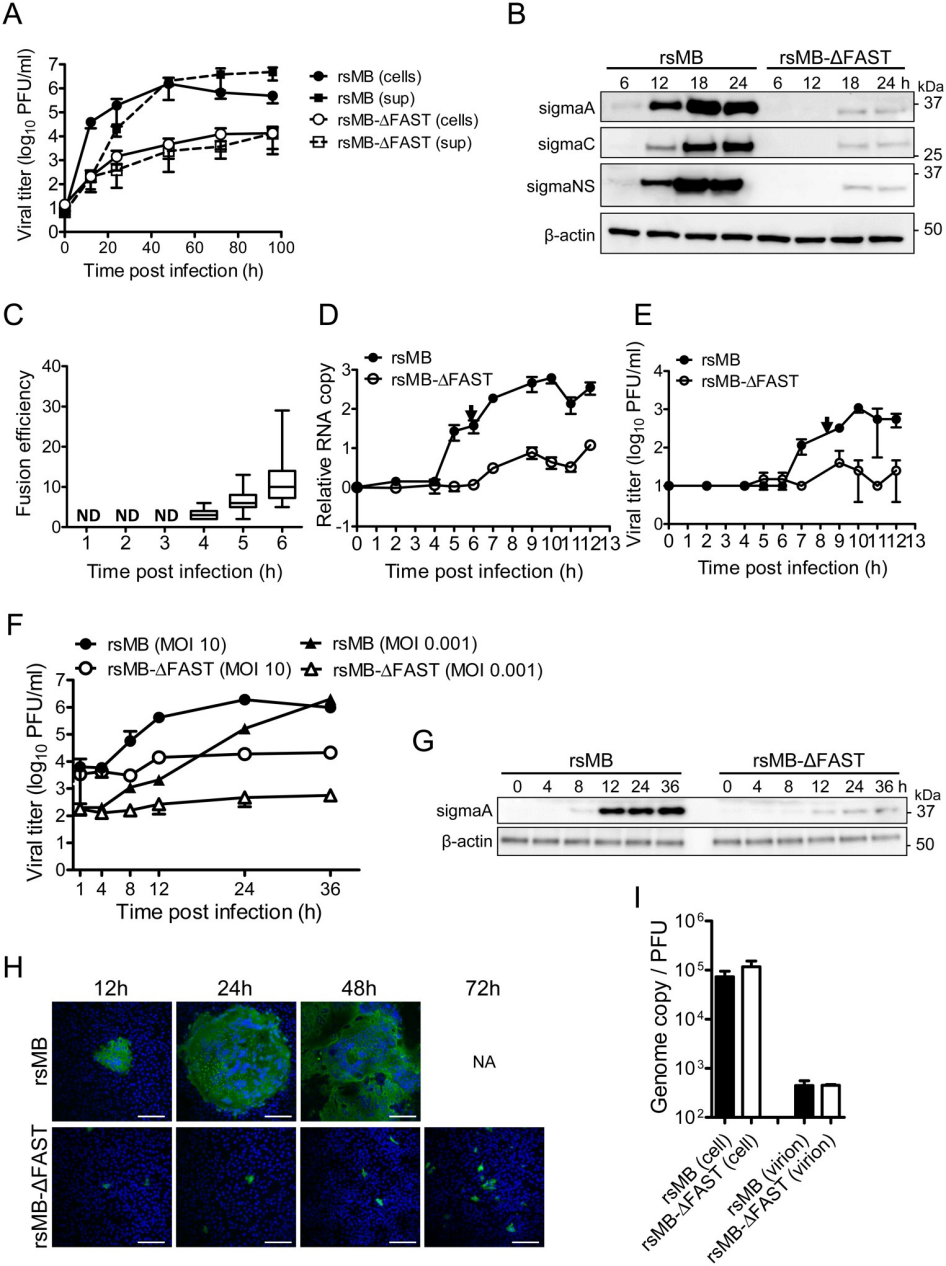
FAST protein enhances viral replication at an early phase of infection. (*A*) Vero cells were infected with rsMB or rsMB-ΔFAST at a multiplicity-of-infection (MOI) of 0.01 plaque-forming units (PFU)/cell. Infectious virus titers in the supernatant and cell lysate were examined. Data are expressed as means ± SD (*n* = 3). (*B*) Time course of viral protein expression. Vero cells were infected with rsMB or rsMB-ΔFAST at a MOI of 0.01 PFU/cell. Whole-cell extracts were subjected to western blotting. Viral proteins were detected with antibodies to sigmaC, p17, and sigmaA. (*C–E*) Vero cells were infected with rsMB or rsMB-ΔFAST at a MOI of 0.01 PFU/cell and incubated for various intervals. (*C*) To determine the time course of syncytium formation, cells were fixed and viral sigmaC antigens were visualized by immunostaining. Numbers of cells involved per syncytium were plotted. Data are presented as a box plot (*n* = 11–30). (*D*, *E*) To determine the kinetics of viral replication, cells were disrupted at indicated times post infection by freeze-thawing. Copy numbers of the genomic RNA L1 gene (*D*) and infectious virus titers (*E*) were investigated by TaqMan quantitative PCR and plaque-formation assay, respectively. Arrows indicate the time points of primary increase of levels of viral genomes and infectious virus particles. NA, not available; ND, not detected. Data are expressed as means ± SD (*n* = 3). (*F*, *G*) Vero cells were infected with rsMB or rsMB-ΔFAST at a MOI of 0.001 or 10 PFU/cell. (*F*) Virus infectious titers were determined by the plaque assay. (*G*) Viral antigens in whole-cell extracts of Vero cells infected with rsMB or rsMB-ΔFAST at a MOI of 10 PFU/cell were detected with an anti-sigmaA antibody. An anti-β-actin antibody was used as a loading control. (*H*) Time course of virus spread in monolayers of Vero cells. Vero cells were infected with rsMB or rsMB-ΔFAST at a MOI of 0.01 PFU/cell. After virus adsorption at 37°C for 1 h, cells were overlaid with 0.8% agarose gel and incubated. At the indicated time points post infection, cells were fixed and viral antigens were detected by immunostaining with murine anti-PRV-MB serum followed by anti-mouse IgG-CF488. NA, not available. Scale bars are 200 μm. (*I*) Ratio between the viral genome copy number and the infectious virus titer in whole-cell lysates and purified virions. The genome copy number of positive-stranded L1 gene segments and the number of plaque-forming units of purified rsMB and rsMB-ΔFAST were calculated. Data are expressed as means ± SD (*n* = 3) and were statistically analyzed using the *t*-test.

The rescue of replication-competent FAST-p10-deficient viruses demonstrated that cell–cell fusion activity of FAST-p10 was not essential for viral replication. We next investigated the replication kinetics of rsMB and rsMB-ΔFAST. The growth rate of rsMB-ΔFAST was significantly lower than that of rsMB in Vero cells (Fig 2A and 2B) and in human cell lines (S2A Fig), demonstrating that FAST-p10 has a role in enhancement of viral replication. We examined the replication and cell–cell fusion kinetics of rsMB and rsMB-ΔFAST at immediate–early time points. In rsMB-infected cells, cell–cell fusion appeared as early as 4 h after viral infection, and syncytial size increased during viral replication (Fig 2C and S2B Fig). The amounts of positive-stranded viral RNA in rsMB-infected cells increased suddenly at 5 h post infection, whereas those in rsMB-ΔFAST-infected cells were lower, with only a gradual increase (Fig 2D). The rsMB infectious virus titer increased at 7 h post infection and continued to increase until 10 h post infection, whereas the rsMB-ΔFAST infectious virus titer remained low (Fig 2E). With Vero cells infected with rsMB at a multiplicity-of-infection (MOI) of 0.01 plaque-forming units (PFU)/cell, infectious viruses were identified in the culture supernatant at 15 h post infection (S2C Fig). Transfection of cells with a FAST-p10 expression plasmid did not affect cell viability up to 30 h post transfection (S2D Fig), suggesting that increased levels of infectious viruses in rsMB-infected cells did not result from newly synthesized virion released from disrupted cells. When cells transfected with FAST-p10 expression plasmid (or mock-transfected) were subsequently transfected with purified mRNA encoding secretory NanoLuc luciferase (NLuc), expression of FAST-p10 did not affect NLuc activity, indicating that FAST-p10 did not affect cap-dependent translation (S2E Fig). To examine whether cell–cell fusion promotes the spread of progeny virions, single-step replication kinetics at a high MOI were investigated. At a MOI of 10 PFU/cell (as well as 0.01 PFU/cell), the titer of rsMB increased markedly at 8 h post infection, while the titer of rsMB-ΔFAST only increased gradually (Fig 2F and 2G).

To examine the time courses of the spread of rsMB and rsMB-ΔFAST, a monolayer of Vero cells was infected with each virus and overlaid with agarose gel. Immunostaining with specific antibodies revealed that viral antigens in rsMB-infected cells spread efficiently and syncytium formed. By contrast, the spread of rsMB-ΔFAST was restricted (Fig 2H). Even at 72 h post infection, only tiny foci were observed in rsMB-ΔFAST-infected cells. These results suggest that cell–cell fusion promoted release of rsMB virions in the late stage of infection, while release of rsMB-ΔFAST virions was restricted due to the lack of FAST protein. To examine whether rsMB-ΔFAST produced deficient virus particles, we examined the ratio between the viral genome copy number and the infectious virus titer for rsMB and rsMB-ΔFAST. In whole-cell lysates and purified virus particles, this ratio was similar for rsMB and rsMB-ΔFAST (Fig 2I), indicating that rsMB-ΔFAST produced intact virions as efficiently as rsMB.

### Cell–cell fusion activity of FAST protein enhances viral replication

**Fig 3:**
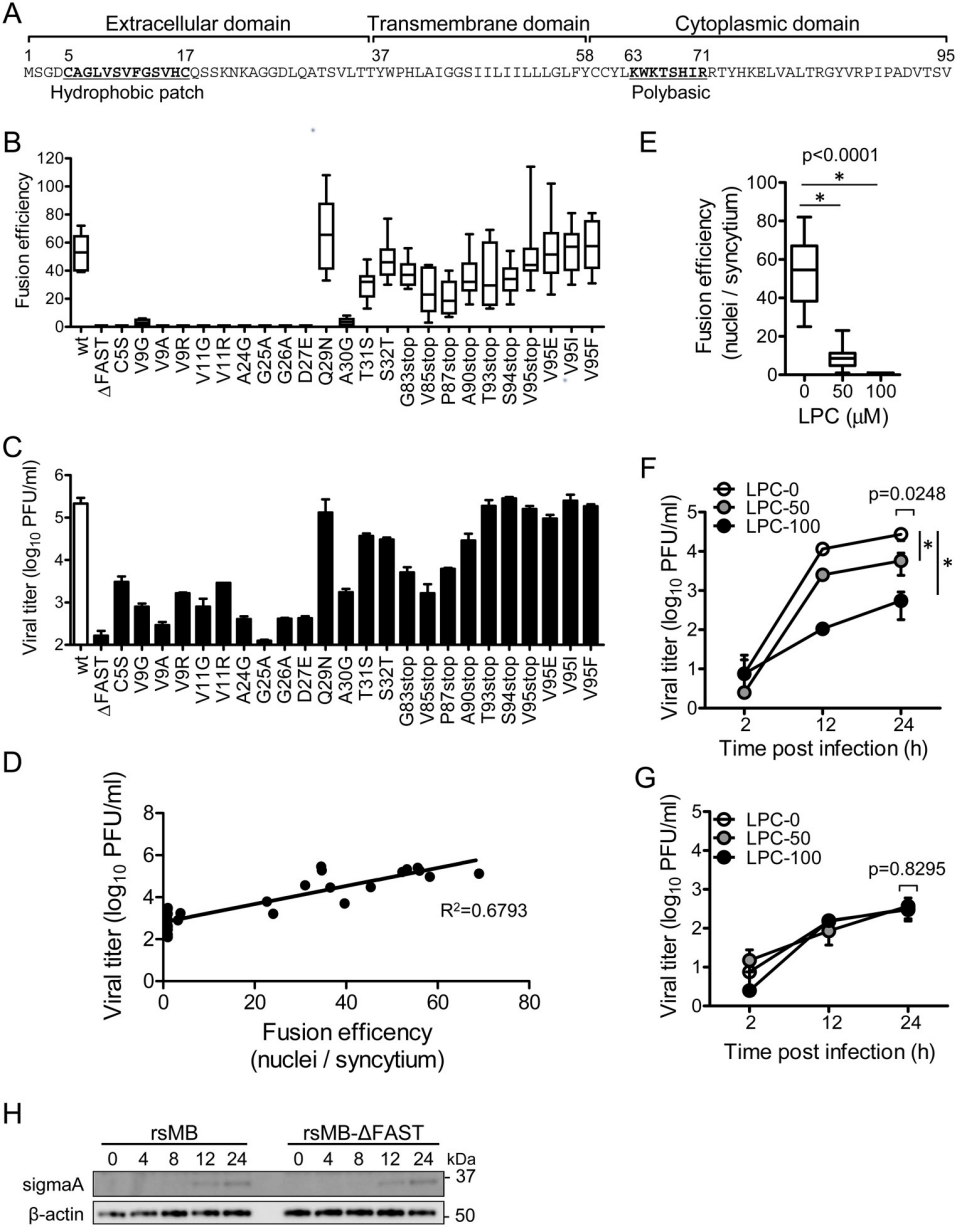
Cell–cell fusion activity correlates with virus propagation. (*A*) Deduced amino acid sequences of pteropine orthoreovirus (PRV) strain MB (rsMB) FAST-p10 protein. Functional domains and peptide motifs are indicated [2]. (*B*) Vero cells were infected with the mutant PRV-MB strains, which harbored mutant FAST-p10 genes with single amino acid substitutions or C-terminal truncations. At 16 h post infection, the syncytium-forming activity of each mutant was determined by calculating the number of nuclei involved per syncytium Data are presented as a box plot (*n* = 8–14). (*C*) Propagation of FAST-p10 mutant viruses. Vero cells were infected with rsMB or FAST-p10 mutant viruses at a multiplicity-of-infection (MOI) of 0.01 plaque-forming units (PFU)/cell and incubated for 72 h. Infectious virus titers in cell lysates were determined. Data are expressed as means ± SD (*n* = 3). (*D*) Correlation of syncytium-forming activity and virus propagation, with a linear regression line. Mean values of fusion activity (*B*) and viral replication (*C*) were plotted on the x- and y-axis, respectively. (*E–G*) Lysophosphatidylcholine (LPC) impaired replication of rsMB, but not of the FAST-p10-deficient mutant rsMB-ΔFAST. (*E*) Vero cells were infected with rsMB or rsMB-ΔFAST at a MOI of 0.01 PFU/cell. At 2 h post infection, cell-culture medium was replaced with medium containing LPC (0–100 μM). Cells were fixed at 16 h post infection, and virus-infected foci were visualized by immunostaining for sigmaC. Fusion efficiency was determined by the number of nuclei involved per syncytium. Data are presented as a box plot and were statistically analyzed using the *t*-test (*n* = 14–26). (*F*–*G*) Vero cells were infected with (*F*) rsMB or (*G*) rsMB-ΔFAST at a MOI of 0.01 PFU/cell. At 2 h post infection, culture medium was replaced with medium containing LPC (0–100 μM). Infectious virus titers in cell lysates were determined by plaque-formation assay. Data are expressed as means ± SD (*n* = 3). * indicates *p* < 0.05 (Dunnett’s multiple comparison test). (H) Time course of viral protein expression. Vero cells were infected with rsMB or rsMB-ΔFAST at a MOI of 0.1 PFU/cell. Viral antigens in whole-cell extracts were detected with an anti-sigmaA antibody. An anti-β-actin antibody was used as a loading control.

PRV FAST-p10 protein has a simple structure including an N-terminal extracellular domain, a transmembrane domain, and a C-terminal intracellular domain (Fig 3A) [2]. To investigate the relationship between fusion activity of FAST-p10 and viral replication, a series of mutant viruses encoding FAST-p10 variants with variable fusion activities were generated by single amino acid substitutions or truncation of the C-terminal region. Mutations in the S1 gene segment of the FAST-p10 ORF were confirmed by sequencing analysis of cDNAs generated from viral genomic RNA of FAST-p10 mutant viruses. Most single amino acid substitutions within the N-terminal ectodomain, including substitutions within the hydrophobic patch, abolished cell–cell fusion activity, whereas truncations of the C-terminal region affected, but did not abolish, fusion activity (Fig 3B and S3A Fig). With these mutants, virus propagation and cell–cell fusion activities showed positive linear correlation in Vero cells (Fig 3C and 3D) and human embryonic kidney 293T cells (S3B Fig).

Lysophosphatidylcholine (LPC) is a minor phospholipid component of cell plasma membranes and inhibits membrane fusion induced by enveloped viruses and FAST proteins [32,33]. Syncytium formation induced by PRV infection was inhibited by addition of LPC in a dose-dependent manner, and was completely abolished with 100 μM LPC (Fig 3E and S3C Fig). Replication of rsMB decreased with increasing LPC concentration (Fig 3F), whereas replication of rsMB-ΔFAST was not affected by the presence of LPC (Fig 3G). At 100 μM LPC, replication of rsMB was comparable to that of rsMB-ΔFAST.

The time course of protein expression for rsMB and rsMB-ΔFAST was similar in the presence of 100 μM LPC (Fig 3H). These results indicate that viral replication and protein expression was enhanced by cell–cell fusion activity of FAST protein. We confirmed these results using the lymphoid-like murine sarcoma S180-Meiji cell line (S3D Fig). S180-Meiji cells did not exhibit cell–cell fusion upon rsMB infection (S3E Fig). The growth kinetics of rsMB and rsMB-ΔFAST revealed by plaque assay and western blotting were similar in S180-Meiji cells (S3F and S3G Fig), indicating that cell–cell fusion was required for increased viral protein expression and infectious virus production.

### Enhancement of viral replication by exogenous syncytium formation by recombinant FAST-p10 expression

**Fig 4:**
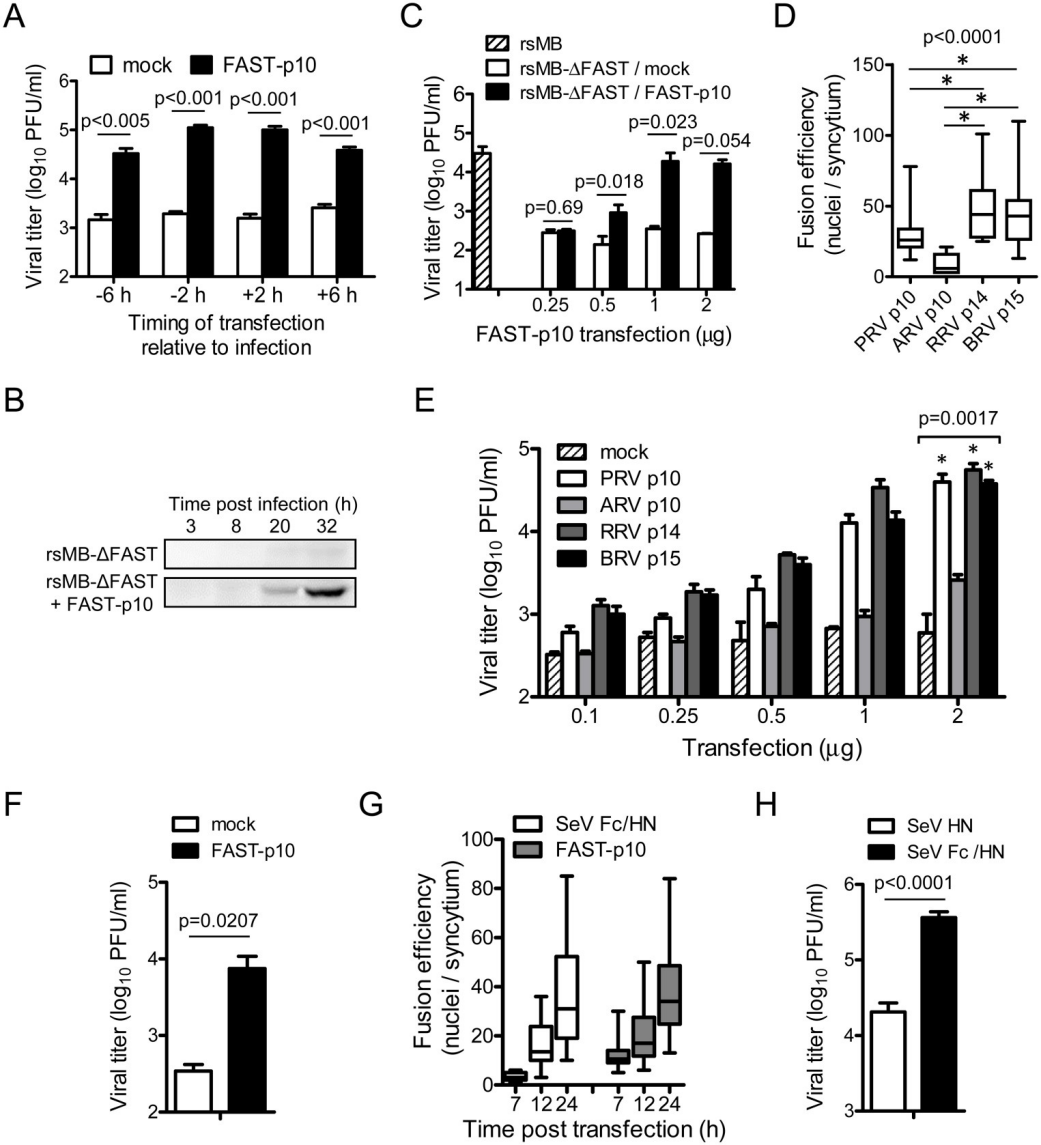
Cell–cell fusion by FAST proteins enhances replication of pteropine orthoreovirus (PRV). (*A*) Enhancement of viral replication by recombinant PRV FAST-p10 protein. Vero cells were transfected with FAST-p10 expression vector at the indicated times either before or after infection with a FAST-p10-deficient PRV mutant (rsMB-ΔFAST). Infectious virus titers in cell lysates at 16 h post infection were determined. Data are expressed as means ± SD (*n* = 3) and were statistically analyzed using the *t*-test. (*B*) Whole-cell lysates of Vero cells transfected with FAST-p10 expression vector or empty vector 2 h before infection with rsMB-ΔFAST were prepared, and viral sigmaC protein was detected by western blotting. (*C*) Vero cells were transfected with FAST-p10 expression vector or empty vector (0.25–2.0 μg/well) 2 h before infection with wild-type PRV (rsMB) or rsMB-ΔFAST. Infectious virus titers in cell lysates at 16 h post infection were determined. Data are expressed as means ± SD (*n* = 3) and were statistically analyzed using the *t*-test. (*D*) Efficiency of syncytium formation by different members of the FAST protein family. Vero cells were transfected with recombinant expression vectors (0.5 μg/well) for PRV FAST-p10, avian orthoreovirus (ARV) FAST-p10, reptilian orthoreovirus (RRV) FAST-p14, or baboon orthoreovirus (BRV) FAST-p15, or with empty vector. At 16 h post infection, cells were fixed and numbers of nuclei per syncytium were counted (*n* = 5–33). * indicates *p* < 0.05 (Dunn’s multiple-comparison test). (*E*) Vero cells were transfected with expression vectors for PRV FAST-p10, ARV FAST-p10, RRV FAST-p14, or BRV FAST-p15, or with empty vector (0.1–2.0 μg/well) 2 h before infection with rsMB-ΔFAST. Infectious virus titers in cell lysates at 16 h post infection were determined. Data are expressed as means ± SD (*n* = 4). Viral titers in cells with 2.0 μg/well plasmid transfections were statistically compared with titers in mock-transfected samples. * indicates *p* < 0.05. (*F*) QT6 cells were transfected with 2 μg of PRV FAST-p10 expression vector or empty vector, followed by infection with ARV at a MOI of 0.001 PFU/cell. Infectious virus titers in cell lysates at 16 h post infection were determined and statistically analyzed using the *t*-test. Data are expressed as means ± SD (*n* = 3). (*G*) Time course of syncytium formation by PRV FAST-p10 and modified recombinant Sendai virus (SeV) F (Fc) proteins. Vero cells were transfected with expression vectors either for FAST-p10 or for SeV Fc and SeV HN (0.5 μg each/well). At indicated times post transfection, cells were fixed and numbers of nuclei involved per syncytium were counted. Data are presented as a box plot (*n* = 7–30). (*H*) Enhancement of viral replication by recombinant SeV Fc protein. Vero cells were transfected with expression vectors for SeV Fc and SeV HN proteins (1 μg each/well) 2 h before infection with rsMB-ΔFAST at a MOI of 0.001 PFU/cell. At 16 h post infection, infectious virus titers in the cell lysates were determined. Data are expressed as means ± SD (*n* = 3) and were statistically analyzed using the *t*-test.

To develop a complementation assay for studies of FAST protein-dependent replication, Vero cells were transfected with FAST-p10 expression plasmid before or after infection with rsMB-ΔFAST. Provision of FAST-p10 *in trans* rescued a substantial level (15–64-fold) of rsMB-ΔFAST growth (Fig 4A and 4B). Co-expression of FAST-p10 in rsMB-ΔFAST-infected cells resulted in the formation of syncytium with similar expression of PRV antigens to that found in rsMB-infected cells (S4A Fig). FAST-p10 expression enhanced viral replication in a dose-dependent manner (Fig 4C). Furthermore, a complementation assay was conducted in which cells were transfected with the FAST-p10 expression vector and infected with rsMB-ΔFAST at different MOIs (10, 1, 0.1, and 0.01). Overexpression of FAST-p10 enhanced virus production at all MOIs tested (S4B Fig). The result obtained at a high MOI suggests that enhancement of virus production by FAST-p10 was not due to spread of progeny virions via cell–cell fusion, consistent with the replication kinetics of rsMB and rsMB-ΔFAST at low and high MOIs.

FAST proteins from different *Orthoreovirus* and *Aquareovirus* species have common topologies (ecto-, transmembrane-, and endo-domains), but varying levels of amino acid sequence similarity (S5 Fig) [2]. To determine whether FAST proteins have evolved with species-specific functions, we examined the effects of exogenous FAST protein expression in rsMB-ΔFAST propagation. Expression of recombinant RRV FAST-p14 produced the strongest fusion activity, followed by BRV FAST-p15, PRV FAST-p10, and ARV FAST-p10, in transfected Vero cells (Fig 4D). Expression of all recombinant FAST proteins enhanced replication of rsMB-ΔFAST, suggesting that FAST proteins can enhance PRV replication by species-independent mechanisms (Fig 4E). The level of viral propagation reflected the fusion activities of the FAST proteins, with the highest level of viral replication in cells expressing RRV FAST-p14, and the lowest level associated with ARV FAST-p10. The fusion-inducing activity of ARV FAST-p10 was lower than that of other FAST proteins; therefore, we repeated the complementation assay using a higher amount of the ARV FAST-p10 expression plasmid. Transfection of 2.0 or 4.0 μg of the ARV FAST-p10 expression plasmid at 2 h before infection of rsMB-ΔFAST enhanced viral replication, while transfection of 1.0 μg of this plasmid did not (S4C Fig). Syncytium formation induced by ARV FAST-p10 was slower than that induced by other FAST proteins; therefore, the incubation time between transfection and infection was varied. Transfection of 1.0 μg of ARV FAST-p10 expression plasmid at 8 h before infection of rsMB-ΔFAST significantly enhanced viral replication (S4D Fig). These results indicate that ARV FAST-p10 also enhanced replication of rsMB-ΔFAST, although optimized experimental conditions were required due to the lower fusion-inducing activity of this protein.

Notably, the replication of ARV was also enhanced by expression of PRV FAST-p10, indicating that, in addition to PRV, other fusogenic reoviruses are sensitive to FAST protein enhancement (Fig 4F). These results suggest that there is no specificity between FAST proteins and their source viruses, and that fusion activity alone is important for enhancement of viral replication.

To better understand the enhancement of viral replication by cell–cell fusion, we examined the effect of fusogenic F glycoprotein, a class I viral fusion protein derived from Sendai virus (SeV) of the *Paramyxoviridae* family [19,34]. In SeV, the precursor F0 protein is activated by trypsin cleavage at a site located between the F1 and F2 proteins, and further conformational change by HN protein is required for viral envelope–cell or cell–cell fusion activity. To avoid the addition of trypsin, two additional protease cleavage sites were introduced, as described previously, to produce a SeV Fc protein that could induce cell–cell fusion without exogenous protease [35]. Co-expression of SeV Fc and HN induced the formation of syncytium that was morphologically similar to the syncytium induced by FAST-p10 protein at 12 and 24 h post transfection (Fig 4G and S4E and S4F Fig). Immunostaining showed that expression of FAST-p10 or SeV Fc–HN prior to infection with rsMB-ΔFAST resulted in the formation of viral-antigen-positive syncytia similar to those seen in rsMB-infected cells (S4G Fig). Expression of SeV Fc–HN caused a significant increase of 17.8-fold in rsMB-ΔFAST replication at 16 h post infection, compared with expression of SeV HN only (Fig 4H). We were concerned that altered expression of p17 and sigmaC proteins of rsMB-ΔFAST (Fig 1D) affected viral replication; therefore, we performed the complementation assay using p17 and sigmaC expression plasmids. Overexpression of p17 or sigmaC did not enhance replication of rsMB-ΔFAST, indicating that the absence of FAST-p10 expression was responsible for the impairment of viral replication (S4H, S4I and S4J Fig).

### Effects of FAST protein in non-fusogenic-virus replication

**Fig 5:**
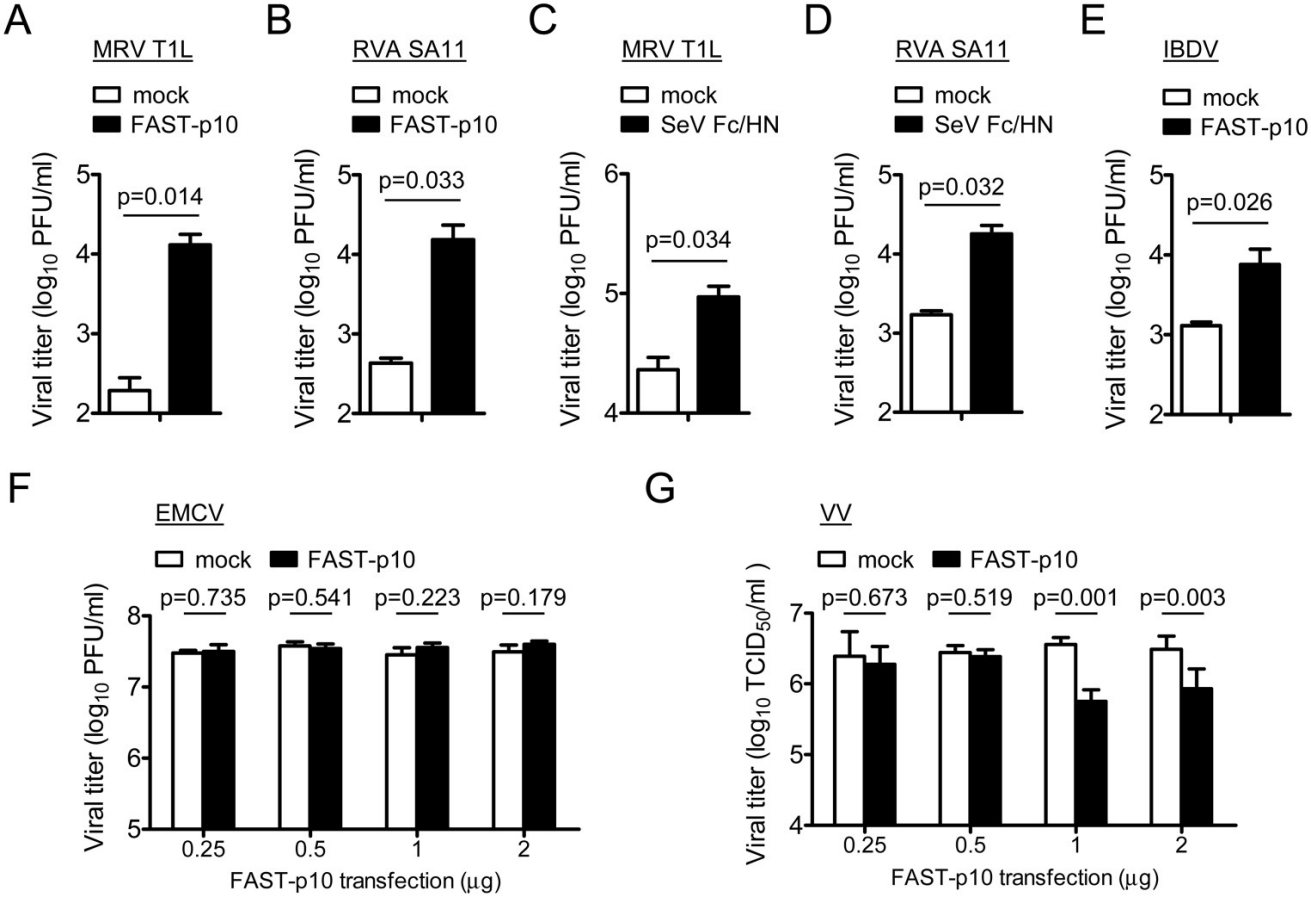
Cell–cell fusion has heterogeneous effects on replication of different viruses. (*A–D*) Effects of cell–cell fusion on replication of non-fusogenic mammalian orthoreovirus (MRV) and group A rotavirus (RVA). (*A*, *B*) Vero cells were transfected with pteropine orthoreovirus (PRV) FAST-p10 expression plasmid vectors or empty vector (1 μg/well). (*C*, *D*) BSR cells were transfected with Sendai virus (SeV) modified recombinant F (Fc) and HN expression plasmid vectors or empty vector (1 μg each/well). At 2 h post transfection, cells were infected with MRV strain T1L (*A*, *C*) or RVA strain SA11 (*B*, *D*) at a multiplicity-of-infection (MOI) of 0.001 plaque-forming units (PFU)/cell. Infectious virus titers in cell lysates at 16 h post infection were determined by plaque-formation assay. Data are expressed as means ± SD (*n* = 3) and were statistically analyzed using the *t*-test. (*E–G*) Effects of cell–cell fusion by PRV FAST-p10 on replication of (*E*) infectious bursal disease virus (IBDV) in DF1 cells, (*F*) encephalomyocarditis virus (EMCV) in Vero cells, and (*G*) vaccinia virus (VV) in BSR cells. Monolayers of DF1, Vero, or BSR cells were transfected with FAST-p10 expression plasmid vectors or empty vector 4 h (*E*) or 2 h (*F*, *G*) before viral infection at a MOI of 0.001 PFU/cell (E–F) or 0.001 TCID_50_/cell (*G*). At 14 h post infection (IBDV), 12 h post infection (EMCV), and 20 h post infection (VV), infectious virus titers were determined. Data are expressed as means ± SD (*n* = 3) and were statistically analyzed using the *t*-test. *p* < 0.05 was considered statistically significant.

We investigated whether FAST protein could enhance replication of viruses that do not encode fusogens. Expression of PRV FAST-p10 protein *in trans* significantly enhanced replication of other *Reoviridae* viruses, including MRV (63-fold) and RVA (35-fold), in Vero cells, relative to mock-transfected cells (Fig 5A and 5B). These observations were consistent with those of a previous study [27]. As expected, SeV F protein also enhanced replication of MRV (4.0-fold) and RVA (9.3-fold) (Fig 5C and 5D). The enhancement of RVA replication by FAST-p10 protein was observed in the absence of trypsin, which is required for activation of newly synthesized RVA virion by cleavage of capsid VP4 protein into VP5 and VP8 fragments [36], suggesting that enhancement of viral replication was not the result of secondary infection by released viruses. Notably, Vero cells are resistant to replication of RVA SA11 strain in the absence of exogenous FAST protein (S6 Fig).

FAST protein-dependent enhancement of viral replication was further investigated in viruses from different families, including infectious bursal disease virus (IBDV, family *Birnaviridae*, two-segment dsRNA genome), encephalomyocarditis virus (EMCV, family *Picornaviridae*, positive-sense single-stranded (ss)RNA genome), and vaccinia virus (VV, family *Poxviridae*, dsDNA genome). Cell–cell fusion induced by FAST-p10 protein enhanced replication of IBDV ~5.5-fold (Fig 5E). In common with reoviruses, IBDV is a segmented dsRNA virus, but whereas reoviruses maintain a transcriptional core particle that contains the dsRNA genome and RNA polymerase complex, the dsRNA of IBDV forms a transcriptionally active ribonucleoprotein complex with RNA-dependent RNA polymerase VP1 and RNA-binding polypeptide VP3 [37]. In contrast to IBDV, replication of EMCV and VV was not enhanced by FAST-p10 protein (Fig 5F and 5G); expression of FAST-p10 protein did not affect replication of EMCV and reduced that of VV in a dose-dependent manner. In summary, FAST protein enhanced replication of dsRNA viruses belonging to the families *Reoviridae* and *Birnaviridae*, but not of viruses belonging to the families *Picornaviridae* and *Poxviridae*.

### FAST-p10 protein is important for PRV pathogenesis

**Fig 6:**
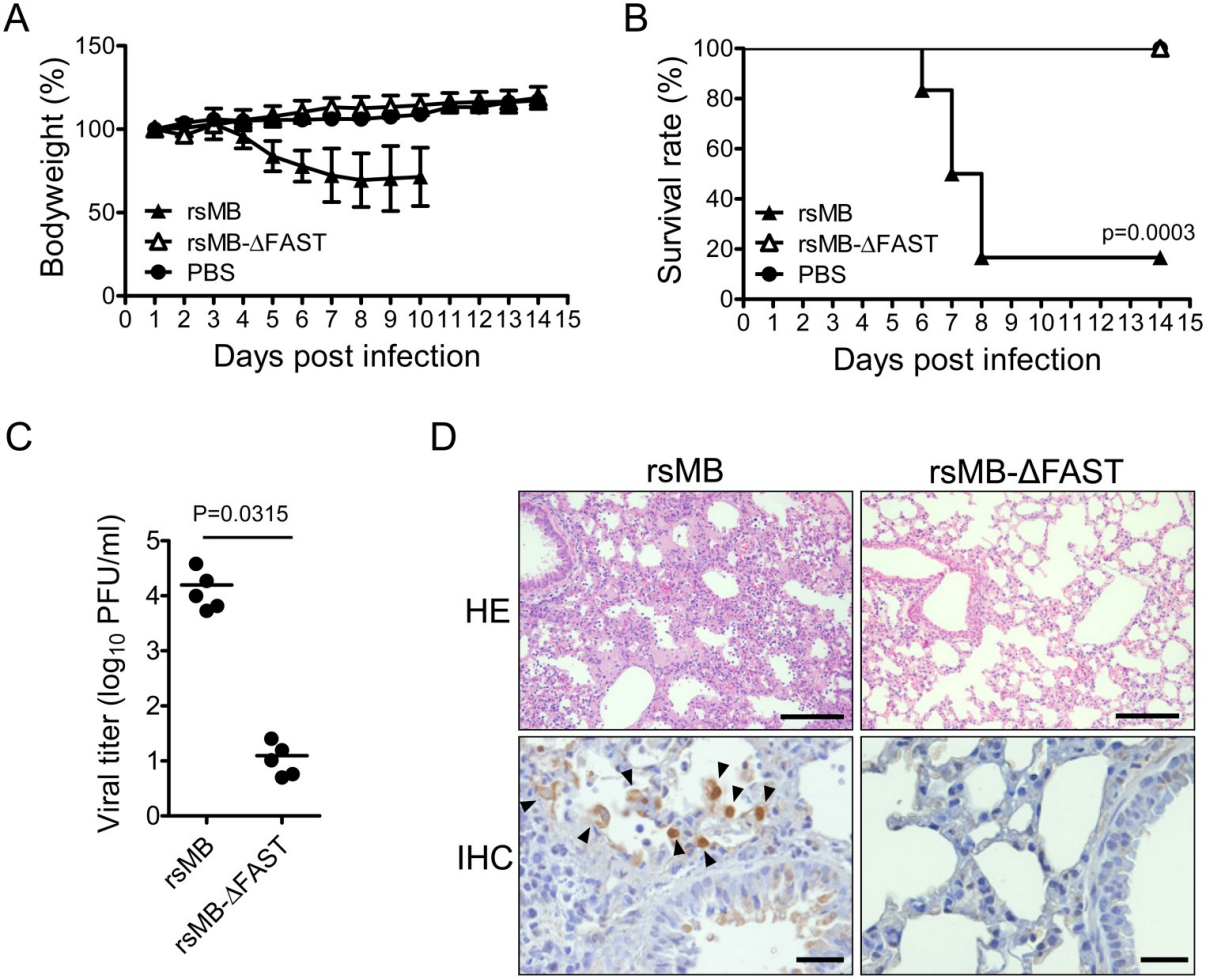
FAST protein is a determinant of pteropine orthoreovirus (PRV) pathogenicity. (*A*, *B*) C3H mice (male, 3-week-old, *n* = 10/group) were intranasally infected with 4 × 10^5^ plaque-forming units (PFU) of wild-type (rsMB) or FAST protein-deficient (rsMB-ΔFAST) PRV. The mice were monitored for (*A*) bodyweight changes and (*B*) survival rate for 14 days following virus inoculation. Survival curves were statistically compared with that in the control group using the log rank test. (*C*) Replication of PRV in mouse lungs. C3H mice (male, 3-week-old, *n* = 5) were intranasally infected with 4 × 10^5^ PFU of rsMB or rsMB-ΔFAST. Animals were euthanized at 5 days post infection. Infectious virus titers in lung homogenates were determined by plaque-formation assay. Virus infectious titers were statistically compared between the two groups using the *t*-test. (*D*) Histopathological examination of C3H mice infected with PRV. Lungs of C3H mice 7 days after infection with rsMB or rsMB-ΔFAST were subjected to histopathological examination by hematoxylin–eosin (HE) staining and immunohistochemistry (IHC) with anti-sigmaC serum. Arrowheads indicate sigmaC-positive cells in IHC. Original magnifications: 200× (HE) and 400× (IHC). Scale bars indicate 100 μm (HE) or 20 μm (IHC).

To investigate the role of FAST-p10 protein *in vivo*, the pathogenicity of wild-type rsMB and rsMB-ΔFAST was compared in a mouse model for lethal PRV infection [38]. Intranasal infection with rsMB caused lethal lung infection (with progressive bodyweight loss in the early stages) in inbred C3H/HeNCrl mice, with 80% mortality within 14 days post infection (Fig 6A and 6B). By contrast, all the mice in the group infected with rsMB-ΔFAST survived, with no bodyweight loss. Viral titers in the lungs of mice infected with rsMB-ΔFAST were low, whereas rsMB replicated efficiently and had high viral titers (Fig 6C). Histopathological analysis of lungs collected on day 5 post infection revealed that rsMB infection resulted in severely damaged lungs with excessive infiltration of lymphocytes and macrophages (Fig 6D). Viral antigens were found in epithelial cells of bronchioles and lung alveolar cells. By contrast, lungs of mice infected with rsMB-ΔFAST were not apparently damaged. These results suggested that FAST-p10 protein enhanced viral replication *in vivo*, contributing to the high pathogenicity of PRV.

### FAST-deficient mutant viruses as attenuated vaccine candidates

**Fig 7:**
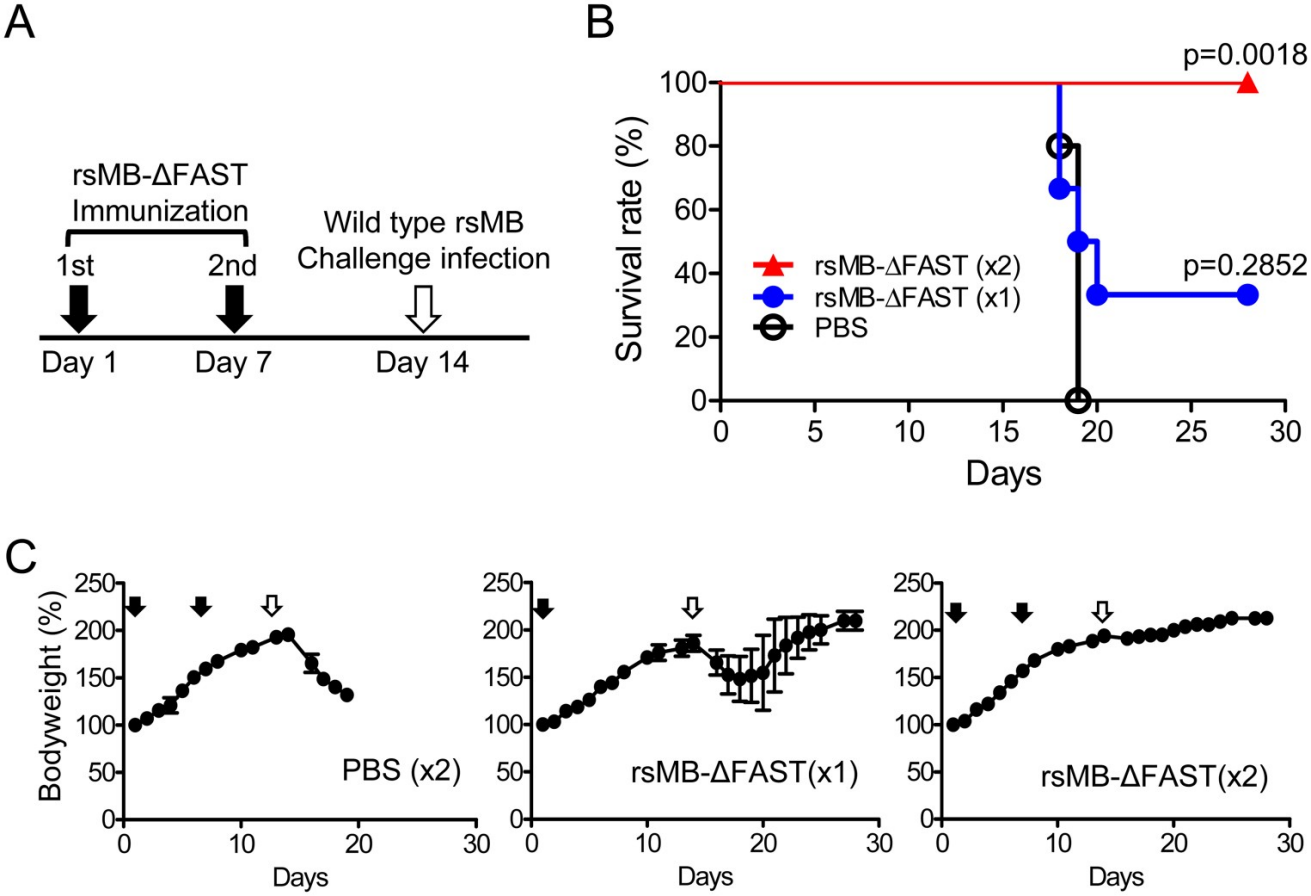
Use of FAST-p10-deficient virus as live attenuated vaccine. (*A*) Schedule of immunization and challenge infection. Male, 3-week-old C3H mice (*n* = 6/group) were intranasally infected with 4 × 10^5^ plaque-forming units (PFU) of FAST-p10-deficient pteropine orthoreovirus (PRV) mutant (rsMB-ΔFAST) either once (on day 1) or twice (on day 1 and day 7). Control mice were intranasally inoculated with phosphate-buffered saline (PBS). On day 14, mice were intranasally infected with a lethal dose of wild-type PRV (rsMB, 4 × 10^5^ PFU). Animals were monitored daily for 14 days after challenge infection. (*B*) Survival curves of non-infected mice and mice inoculated with one dose (×1) or two doses (×2) of rsMB-ΔFAST, or with PBS, and challenged with rsMB. Survival curves were statistically compared with that in the control group using the log rank test. (*C*) Bodyweight changes in mice inoculated with one dose (×1) or two doses (×2) of rsMB-ΔFAST, or with PBS, and challenged with rsMB. Black arrows indicate immunization on day 0 or day 7; white arrows indicate challenge infection on day 14.

The attenuation of FAST-deficient PRV in the mouse model suggested that FAST-deficient mutant viruses were promising candidates for the development of fusogenic-reovirus vaccines. To develop a live attenuated vaccine to protect host species against pathogenic PRV, C3H mice were infected intranasally with 2.0 × 10^5^ PFU per animal of rsMB-ΔFAST either once or twice (with a 1 week interval between infections) (Fig 7A). Control mice were intranasally inoculated with PBS. On day 14 after the first inoculation, mice were challenged with a lethal dose (4.0 × 10^5^ PFU per animal) of rsMB intranasally. Control mice challenged with rsMB underwent progressive loss of bodyweight, with 100% mortality by day 5 post infection (Fig 7B and 7C). Mice immunized with a single dose of rsMB-ΔFAST underwent initial loss of bodyweight, with 67% mortality by day 6 post infection, but 33% of these mice survived and recovered, regaining the lost bodyweight (Fig 7B and 7C). Mice immunized with two doses of rsMB-ΔFAST were protected against rsMB, with 100% survival and no loss of bodyweight (Fig 7B and 7C). Similar results were obtained by immunization with rsMB-FAST/A30G, a mutant with a single amino acid substitution in FAST-p10, resulting in impaired fusion activity and replication (Fig 3B and 3C and S3A Fig). Mice immunized with a single dose of rsMB-FAST/A30G had a 33% survival rate 14 days after rsMB challenge, and surviving mice recovered and regained lost bodyweight (S7A and S7B Fig). Mice immunized with two doses of rsMB-FAST/A30G were protected against rsMB, with 100% survival (S7A Fig). The results demonstrated that attenuation of the cell–cell fusion induction activity of FAST protein is a novel strategy to develop live attenuated vaccine strains for fusogenic reoviruses, including PRV and ARV.

## Discussion

The FAST protein family consists of the only fusogenic proteins that have been found in non-enveloped viruses. Although the molecular biology of several recombinant FAST proteins, including PRV FAST-p10, ARV FAST-p10, RRV FAST-p14, BRV FAST-p15, and *Aquareovirus* FAST-p16 and FAST-p22, has been extensively studied [1,3,4,6,8], the functions of FAST proteins in viral replication have not previously been determined. By studying PRV mutants with deletion or alteration of FAST-p10, we have now clearly shown that FAST proteins can act as enhancers of viral replication and virulence. Using a PRV reverse genetics system [28], we generated FAST protein-deficient PRV, which was replication-competent, but which had significantly lower replication efficiency than wild-type PRV, indicating that cell–cell fusion enhanced viral replication. In the first 12 h after rsMB infection, we first observed cell–cell fusion, then enhanced (relative to rsMB-ΔFAST) synthesis of viral genomes and infectious virion production. The release of infectious virus into the culture medium was detected at 15 h post infection, suggesting that enhancement of viral replication was not associated with secondary infection by released viruses. The replication kinetics of rsMB and rsMB-ΔFAST at a high MOI and the results of the complementation assay using the FAST-p10 expression vector at a high MOI suggested that the spread of progeny virions did not contribute to the enhancement of replication by FAST protein.

Syncytium formation by recombinant FAST-p10 protein recovered replication of rsMB-ΔFAST in a dose-dependent manner, demonstrating that impaired replication of FAST-deficient PRV was the result of a lack of cell–cell fusion activity. Similarly, the fusion activities of mutant PRVs with FAST-p10 variants were linearly correlated to enhancement of viral replication, and inhibition of cell–cell fusion by LPC suppressed the replication of rsMB (but not rsMB-ΔFAST) in a dose-dependent manner. FAST proteins from four different reoviruses all enhanced replication of rsMB-ΔFAST, and the level of enhancement for each was related to the cell–cell fusion activity. The finding that replication of rsMB-ΔFAST was enhanced by a paramyxovirus fusion protein supports the hypothesis that replication of PRV-MB largely depends on cell–cell fusion. However, the level of enhancement differed between FAST protein and paramyxovirus F protein (Fig 4A and 4H), suggesting that FAST protein has an unknown function distinct from induction of syncytium formation. These results all confirmed the importance of cell–cell fusion for PRV replication and suggested that drugs or antibodies targeting FAST proteins could be developed to inhibit replication of fusogenic reoviruses.

Unlike PRV, the non-fusogenic reoviruses MRV and RVA were capable of replication to high titers in the absence of syncytium formation. Notably, however, syncytium formation by PRV FAST-p10 enhanced the replication of MRV, RVA, and IBDV, which all have dsRNA genomes, but not replication of EMCV (ssRNA) or VV (dsDNA). Although the replication cycle of PRV has not been studied, speculations can be made based on detailed studies of the replication of MRV and bluetongue virus (BTV), which both belong to the family *Reoviridae*. In MRV, which is genetically close to PRV, viral positive-sense ssRNA is synthesized inside uncoated core particles and then released into the cytoplasm through the pore structures of the core particles, to function as messenger RNA or viral genomic RNA [39]. Notably, we observed enhancement of viral RNA synthesis soon after cell–cell fusion occurred (as early as 5 h post infection), suggesting that cell–cell fusion enhanced primary RNA synthesis within the transcriptionally activated core particle in the cytoplasm. *In vitro* synthesis of BTV RNA has previously demonstrated that the core particle is activated by divalent cations, that it continuously synthesizes ssRNA [40], and that RNA production requires nucleotide triphosphates and S-adenosyl-methionine for polymerase and capping reactions [41]. It is possible that core particles within syncytia are able to synthesize more viral RNA than those in discrete cells because more substrates for viral RNA synthesis are available in syncytia. A similar mechanism may be responsible for the enhancement of recovery of RVA by plasmid-based reverse genetics using FAST protein. Following production of the immature core particle after RVA plasmid transfection, production of RVA genome from the core would be enhanced by cell–cell fusion. To obtain a detailed understanding of the mechanisms involved in replication enhancement, further experiments will be required, focusing on ssRNA synthesis from core particles in syncytia. The difference between the spread of rsMB and rsMB-ΔFAST suggested that progeny viruses were efficiently released via disruption of cells following syncytium formation at a late stage of viral replication. Most non-enveloped viruses lack fusogenic proteins but efficiently replicate and egress. This suggests that a unique mechanism underlying release of progeny virions, which is largely dependent on FAST protein-induced syncytium formation, evolved in fusogenic reoviruses.

In common with reoviruses, IBDV is a non-enveloped virus with a segmented dsRNA genome. However, IBDV lacks the T = 2 shell structure that is present in other dsRNA viruses, in which the dsRNA genome is isolated from host innate immune system sensors. The IBDV dsRNA genome instead forms a transcriptionally active ribonucleoprotein complex [37]. On the basis of its structural characteristics, IBDV is considered to be an evolutionary link between dsRNA and ssRNA viruses [42,43]. Replication of IBDV by FAST-p10 protein was only enhanced ~5.5-fold, compared with 15–64-fold for PRV rsMB-ΔFAST, 63-fold for MRV, and 35-fold for RVA, indicating that syncytium formation does not contribute as much to replication of IBDV as to that of other dsRNA viruses. Further research focusing on the common factors in *Reoviridae* and *Birnaviridae* viruses could help to uncover the mechanisms underlying FAST protein-dependent enhancement of viral replication.

Replication of VV was not enhanced by cell–cell fusion, possibly because VV is coated by an envelope derived from the early endosomes or trans-Golgi network [44], suggesting that FAST protein interfered with this lipid bilayer membrane. Although evidence suggests that cell–cell fusion occurring during the replication of enveloped viruses (such as HIV) would promote cell–cell viral transmission of viruses [45], our result suggests that a high level of syncytium formation can inhibit the replication of enveloped viruses.

In a small-rodent model, we demonstrated that FAST-p10 protein contributed to enhanced virus replication and pathogenesis *in vivo*. The low infectious virus titer of rsMB-ΔFAST in lung (~10 PFU per 100 mg of tissue) indicated that replication and spread of PRV was severely impaired in the absence of FAST protein. By contrast, non-fusogenic MRV replicates and spreads efficiently in murine lung without a fusogenic protein [46]. We did not identify cell–cell fusion in the lungs of rsMB-infected mice by histological examination, suggesting that, if syncytia were present, disruption of the tissues made it difficult to find them in the tissue sections. Careful examination to identify syncytium formation in PRV-infected tissue (e.g., immunostaining of the plasma membrane in PRV-infected tissue sections) may be needed to clarify the role of FAST protein in viral replication and pathogenesis *in vivo*.

Our results demonstrated that rsMB-ΔFAST can be developed as a candidate for an attenuated vaccine strain. Since the first report of PRV infection in humans in 2007, PRV has been detected in humans and bats in eastern and south-eastern Asian countries, suggesting that PRV is a bat-borne zoonotic virus [47]. Patients with PRV infections have a high fever, sore throat, cough, and/or diarrhea. Sero-epidemiological studies conducted in Malaysia and Vietnam reported that anti-PRV antibodies were detected in 4–13% of people [30,48]. Furthermore, the PRV genome was detected in 17% of patients with respiratory symptoms in Malaysia [49]. These studies suggest that PRV infections are common in south-eastern Asian countries and that PRV is an important respiratory virus. The strategy of developing a live attenuated vaccine strain by attenuation of FAST protein can also be applied to ARV, which causes arthritis and tenosynovitis in chickens. A recent epidemiological study of ARV in the USA and Canada reported the emergence of antigenic variant strains that are antigenically distinct from the vaccine strain [50,51]. Although single-dose infection with rsMB-ΔFAST did not protect animals effectively, two-dose inoculation completely protected mice from lethal infection. We assessed two PRV strains as immunogens, with complete (rsMB-ΔFAST) or moderate (rsMB-FAST/A30G) loss of fusion activity, and both strains replicated poorly *in vitro*. However, we produced a number of PRV mutants with different FAST-p10 mutations and different levels of replication *in vitro*, and one of these mutants with moderate replication might provide sufficient induction of neutralizing antibodies with minimal pathogenicity to enable its use as a single-dose immunogen. In this study, challenge infection was performed at 1 week post immunization because animals became resistant to PRV-MB infection over time. FAST-deficient viruses do not replicate efficiently *in vivo*; therefore, establishment of long-lasting immunity is a concern for vaccine development. The kinetics of specific antibodies following FAST-deficient virus infection should be evaluated in a future study. Moreover, as FAST protein is not essential for viral replication, foreign genes could be inserted into the position of the FAST ORF, enabling rsMB-ΔFAST to be developed as an attenuated transduction vector.

The presence of fusogenic viruses in the different genera of reoviruses, including *Orthoreovirus* and *Aquareovirus*, suggests that the fusogenic characteristic was common among the ancestors of modern *Reoviridae* viruses. The FAST protein fusion-dependent mechanism may have conferred an evolutionary advantage on these viruses through enhanced efficiency of replication and transmission. However, this hypothesis raises the question of why only the limited group of reoviruses among all non-enveloped viruses acquired FAST proteins. One possibility is that fusogenic activity is too pathogenic to maintain sustainable host–pathogen relationships without some as-yet-unidentified control mechanism. Viral non-structural proteins support the viral replication cycle by interacting with host factors or other viral proteins, but the function of FAST proteins is dissimilar to known viral non-structural proteins. Thus, FAST protein could be categorized as a novel type of viral non-structural protein.

## Materials and methods

### Cells

Monkey kidney epithelial Vero cells, murine fibroblast L929 cells, 293T cells, and canine kidney MDCK cells (all from American Type Culture Collection, Manassas, VA) were grown in high-glucose Dulbecco’s modified Eagle’s medium (DMEM; Nacalai Tesque, Kyoto, Japan) supplemented with 5% fetal bovine serum (FBS; Gibco, Thermo Fisher Scientific, Waltham, MA, USA). Monkey kidney epithelial MA104 cells were provided by Dr. Hiroshi Ushijima (Nihon University). Quail fibroblast QT6 cells were provided by Dr. Kazuyoshi Ikuta (Osaka University). Chicken fibroblast DF1 cells were provided by Dr. Tsuyoshi Yamaguchi (Tottori University). BSR cells, which are derived from baby hamster kidney cells, were provided by Dr. Polly Roy (London School of Hygiene and Tropical Medicine). MA104, QT6, DF1, and BSR cells were grown in DMEM containing 5% FBS. The U251, CAKI-1, HOP92, UO-31, LOX-IMVI, SF-295, KM12, TK-10, SK-OV-3, DU-145, ADR-RES, H322M, and OVCAR-3 human cancer cell lines were provided by Dr. Toru Okamoto (Walter and Eliza Hall Institute of Medical Research) and were cultured in RPMI1640 (Nacalai Tesque) containing 10% FBS. Murine sarcoma S180-Meiji cells were kindly supplied by the Cell Resource Center for Biomedical Research, Institute of Development, Aging, and Cancer (Tohoku University) and were cultured in DMEM supplemented with 10% FBS.

### Viruses

PRV strain Miyazaki-Bali/2007 (PRV-MB) was isolated from a Japanese patient who had returned from Bali, Indonesia, and been hospitalized with high fever, joint pain, sore throat, and cough [52]. A recombinant strain of PRV-MB (rsMB) generated by reverse genetics was amplified in L929 cells and used as wild-type virus [28]. MRV strain T1L and EMCV were amplified in L929 cells. Viral titers of PRV, MRV, and EMCV were determined by plaque assay using L929 cells. ARV strain 58–132 was amplified in QT6 cells, and viral titer was determined by plaque assay using QT6 cells. IBDV OKYMY strain [53] was amplified in DF1 cells, and viral titer was determined by 50% tissue-culture infectious dose (TCID_50_) using DF1 cells. The recombinant RVA strain SA11 [27] was amplified in MA104 cells with 0.5 μg/ml trypsin, and viral titer was determined by plaque assay as described previously [27]. SeV strain Z [54] was amplified in MDCK cells, and viral titer was determined by plaque assay using MDCK cells. Attenuated recombinant VV expressing T7 RNA polymerase (rDIs-T7pol) was propagated in chick-embryo fibroblasts. Viral titer of rDIs-T7pol was determined by TCID_50_ in BSR cells. Virus stocks were kept at −80°C prior to use.

### Antibodies

Mouse antiserum against PRV-MB sigmaC was generated as described elsewhere [28]. To generate mouse antiserum against recombinant PRV-MB sigmaA and sigmaNS, *Escherichia coli* BL21 cells (Takara, Mountain View, CA, USA) transformed with pTrc-His-MB sigmaA or pTrc-His-MB sigmaNS plasmids were incubated with 1 mM isopropyl β-D-1-thiogalactopyranoside at room temperature for 8 h. Cell pellets were lysed in 1% Triton-X100, and His-tagged viral proteins were purified from the soluble fraction using His-Select R Nickel Affinity Gel (Sigma, St Louis, MO, USA) according to the manufacturer’s instructions. The His-sigmaA or His-sigmaNS fusion protein was mixed with alhydrogel adjuvant 2% (InvivoGen, San Diego, CA, USA) according to the manufacturer’s instructions, and ICR mice (CLEA Japan, Tokyo, Japan) were immunized and boosted with the protein-adjuvant mixture. At 4 weeks after administration of the last booster, whole blood was collected and sera were separated by centrifugation. Antiserum to FAST-p10 was raised in rabbits immunized with peptides corresponding to residues 21–32 (KNKAGGDLQATS) of FAST-p10 (Eurofins Genomics, Ebersberg, Germany). Antiserum to PRV-MB p17 was raised in rabbits immunized with peptides corresponding to residues 125–138 (DDDPEHKRFAIRSI) of PRV-MB p17 (Sigma). Rabbit anti-pan-cadherin antibody (C3678) and anti-mouse IgG-HRP conjugates (A9044) were purchased from Sigma Aldrich). Goat anti-rabbit IgG-CF488 conjugate (20019), goat anti-rabbit IgG-CF594 antibody (20113), and goat anti-mouse IgG CF488 antibody (20018) were purchased from Nacalai Tesque.

### Plasmid Construction

pT7-MB plasmids (L1, L2, L3, M1, M2, M3, S1, S2, S3, and S4) for reverse genetics of PRV-MB were prepared as described previously [28]. PRV-MB-p10 and ARV-p10 genes were amplified by RT-PCR using gene-specific primers, and inserted into the *Bgl*II site of the pCAGGS vector by homologous recombination using In Fusion HD cloning kit (Clontech, Takara) to create pCAG-MBFAST-p10 and pCAG-ARVFAST-p10, respectively. BRV p15 gene (AF406787) and RRV p14 gene (AY238887) were synthesized (Eurofins Genomics) and cloned into pCAGGS plasmid to create pCAG-BRVp15-FAST and pCAG-RRVp14-FAST, respectively. Coding regions of PRV sigmaC, sigmaA, and p17 genes were amplified by RT-PCR and inserted into the *Eco*RI site of pEF-HA plasmid, which was modified from pEF-BOS plasmid using In Fusion HD cloning kit, to create pEF-HA-sigmaC, pEF-HA-sigmaA, and pEF-HA-p17. SeV HN and F genes were amplified by RT-PCR using gene-specific primers and inserted into pEF-BOS vector to create pEF-SeV F and pEF-SeV HN plasmids. To create pEF-SeV Fc plasmid, nucleotides 5′-GCCAACAACAGAGCCAGAAGAGAG, corresponding to amino acids Ara-Asn-Arg-Ara-Arg-Arg-Glu, and 5′-AAGAAAAGGAAAAGA, corresponding to amino acids Lys-Lys-Arg-Lys-Arg, were inserted after Thr101 and Ser115, respectively, in SeV F ORF by site-directed mutagenesis. Secretory NanoLuc luciferase (secNLuc, Promega, Madison, WI, USA) gene was amplified and inserted into T7 plasmid used for reverse genetics by replacing the viral genome with secNLuc gene to generate pT7-secNLuc. To generate recombinant protein expression vectors for protein purification in bacteria, coding regions of MB sigmaA and sigmaNS genes were amplified and cloned downstream of sequences encoding a poly-histidine (His) tag in the pTrc-HisA vector (Life Technologies).

### Generation of mutant plasmids for reverse genetics

Mutant pT7 plasmids for reverse genetics were generated by standard site-directed mutagenesis using KOD-Plus-NEO polymerase (Toyobo, Osaka, Japan) and *Dpn*I restriction enzyme. Mutations encoding single amino acid substitutions in MB FAST-p10 gene were introduced into pT7 rescue plasmids by replacing one or two nucleotides to generate pT7-MBS1-FAST-p10/C5S, /V9G, /V9A, /V9R, /V11G, /V11R, /A24G, /G25A, /G26A, /D27E, /Q29N, /A30G, /T31S, /S32T, /V95E, /V95I, and /V95F. pT7 plasmids encoding truncated FAST-p10 genes were generated with mutations in the S1 gene segment to replace amino acids with a stop codon (TAG), generating pT7-MB S1 p10/G83 stop (TAG at nucleotides 273–275), /V85 stop (279–281), /P87 stop (283–285), /A90 stop (294–296), /T93 stop (303–305), /S94 stop (306–308), and /V95 stop (309–311). The primers used to make FAST-p10 mutants are available upon request.

### Generation of recombinant viruses

The 10 PRV-MB plasmids (L1–S4) for reverse genetics were prepared as previously described [28]. To rescue PRV-MB, monolayers of L929 cells were infected with rDIs-T7pol and incubated at 37°C for 1 h. The culture medium was replaced with DMEM supplemented with 5% FBS, and the cells were transfected with the 10 plasmids using 2 μl of TransIT-LT1 transfection reagent (Mirus Bio, Madison, WI, USA) per 1 μg of plasmid DNA. After incubation at 37°C for 5 days, cells were lysed by freeze-thawing and supernatant was transferred to a monolayer of L929 cells and overlaid with DMEM containing 0.8% agarose (SeaPlaque Agarose, Lonza, Basel, Switzerland). At 4 days post infection, cells were stained with secondary overlay medium containing 0.8% agarose and 0.005% neutral red. Visualized plaques were transferred to L929 cells, and plaque-purified viral clones were amplified and used for experiments.

### Nucleotide sequencing analysis of recombinant viruses

The sequences of all the mutant and wild-type virus stocks used for experiments were confirmed. Briefly, dsRNA genomes were purified from virus stocks using Tri-reagent (Life Technologies). cDNA was synthesized by Thermoscript reverse transcriptase with PRV-MB S1-specific primer that targeted the 3′ UTR. The S1 segment of each virus was then amplified with S1-specific primers (pCAG-S1-F: 5′-CGCGCCGATATCTTAAGATCTGCTTATTTTTGTTCTCAAGT; pCAG-S1-R: 5′-GAGGAGTGAATTCGAAGATCTGATGAATAGCTGTCCTCAG, where underlined nucleotides indicate specific binding sites in the S1 UTRs) and inserted into cloning plasmid using In Fusion HD cloning kit. Nucleotide sequencing was performed with Big Dye terminator (Thermo Fisher Scientific).

To avoid the possibility of contamination with rDIs-T7pol in recombinant PRV-MB stocks, PCR examination was performed using primers specific for the D1R gene of rDIs (forward primer: 5′-TAAGCTAATAGAGCCCGTGAATGC; reverse primer: 5′-CACCTTCTGGTTGCTTTGGTAA). Briefly, DNA was purified from stocks of recombinant MB strains by standard phenol–chloroform extraction. As a control, a plasmid containing the D1R gene from rDIs-T7pol was prepared. Preliminary examination with PrimeSTAR GXL DNA Polymerase and the positive control plasmid demonstrated that the detection limit of the test was 10 ng of DNA/μl.

### Immunostaining of virus-infected cells

To detect viral antigens, PRV-MB-infected cells were fixed with 3.8% formaldehyde and permeabilized with 0.1% Triton X-100. PRV sigmaC antigens were detected by immunostaining with rabbit anti-sigmaC antibody followed by anti-rabbit IgG-CF488. FAST-p10 was detected by immunostaining with rabbit anti-FAST-p10 peptide followed by anti-rabbit IgG CF594 conjugate. For co-staining of the plasma membrane and sigmaC antigen, virus-infected Vero cells were fixed with 100% ethanol. The plasma membrane and sigmaC were visualized by immunostaining with rabbit anti-pan-cadherin antibody and mouse anti-PRV-MB serum followed by anti-rabbit IgG CF594 antibody and anti-mouse IgG CF488 antibody, respectively. To count the numbers of cells per syncytium, Vero cells infected with PRV-MB were fixed in 3.8% formaldehyde at 16 h post infection. After permeabilization with 0.1% Triton X-100, virus-infected foci were detected by immunostaining with mouse anti-PRV-MB serum followed by anti-mouse IgG-HRP conjugates and 3,3-diaminobenzidine tetrahydrochloride hydrate (D5637; Sigma). In immunofluorescence assays, nuclei were stained with DAPI, which was included in VECTASHIELD mounting media (H-1500, Vector Laboratories, Inc.).

### Western blotting

Vero cells were infected with rsMB or rsMB-ΔFAST at MOIs of 0.01–10 PFU/cell. At 24 h post infection, whole-cell extracts were subjected to SDS-polyacrylamide gel electrophoresis. After transfer to a membrane, viral proteins were detected using rabbit anti-sigmaC, rabbit anti-p17, and mouse anti-sigmaA primary antibodies and HRP-conjugated anti-rabbit or anti-mouse antibodies. As a positive control, lysates of 293T cells transfected with plasmid vectors encoding hemagglutinin (HA)-tagged sigmaC, p17, and sigmaA were loaded. Viral proteins were detected with anti-sigmaC, anti-p17, and anti-sigmaA antibodies. HA-tagged recombinant proteins were detected using mouse anti-HA antibody and anti-mouse IgG-HRP conjugate. Western blotting for β-actin was conducted as a loading control. Signals were detected using Pierce Femto Super-Signal reagent (Pierce) and a Luminescent Image Analyzer LAS-3000 (Fujifilm, Tokyo, Japan).

### Inhibitory effect of LPC on viral replication

Confluent monolayers of Vero cells were infected with rsMB or rsMB-ΔFAST at a MOI of 0.01 PFU/cell and incubated at 37°C for 1 h. Cells were washed with PBS four times, which was replaced with DMEM supplemented with 5% FBS and various concentrations (0, 50, or 100 μM) of L-α-LPC (L4129, Sigma Aldrich). At 1, 12, and 24 h post infection, cells were lysed by freeze-thawing and virus titers were determined by plaque assay.

### Effect of FAST protein on viral replication

Confluent monolayers of Vero cells for PRV, MRV, RVA, ARV, and EMCV; BSR cells for rDIs-T7pol; and DF1 cells for IBDV grown in 24-well plates were transfected with 0.25, 0.5, 1, or 2 μg of pCAG-FAST-p10 plasmid or pCAGGS empty plasmid using 2 μl of TransIT-LT1 transfection reagent per 1 μg of plasmid DNA. At 2–12 h post transfection, culture media were replaced with DMEM supplemented with 5% FBS and infected with MRV or RVA at a MOI of 0.001 PFU/cell. After adsorption at 37°C for 1 h, cells were washed with PBS six times and cultured in DMEM with 5% FBS for PRV or FBS-free DMEM for RVA. At 16 h post infection, cells were lysed by freeze–thaw cycles. Lysates of cells infected with RVA were incubated with 10 μg/ml trypsin at 37°C for 30 min before titration. Virus titers were determined by plaque assay except for rDIs-T7pol, for which viral titer was determined by TCID_50_.

### Complementation assay with p17 and sigmaC

Confluent monolayers of Vero cells grown in 24-well plates were transfected with 0.5 or 2 μg of the pCAG-p17 and pCAG-sigmaC plasmids using 2 μl of TransIT-LT1 transfection reagent per μg of plasmid DNA. The pCAGGS empty plasmid was used as a mock transfection control. At 2 h post transfection, culture media were replaced by DMEM supplemented with 5% FBS, and cells were infected with rsMB-ΔFAST at a MOI of 0.01 PFU/cell. After adsorption at 37°C for 1 h, cells were washed with PBS six times and cultured in DMEM with 5% FBS. At 16 h post infection, cells were lysed by freeze-thawing. Virus infectious titers in whole-cell lysates were determined by the plaque assay. Viral antigens in whole-cell lysates were detected by western blotting using rabbit anti-p17, rabbit anti-sigmaC. and mouse anti-sigmaA sera. A mouse anti-β-actin IgG antibody was used as a loading control.

### Virion purification

Whole-cell lysates were clarified by centrifugation at 16,400 ×g for 2 min and the supernatant was collected. Viruses were concentrated by ultracentrifugation in a Beckman SW28 rotor at 89,450 ×g for 2 h. The pellet was resuspended in DMEM and sedimented through CsCl (0.39 g/ml) using a Beckman SW55 rotor at 116,000 ×g for 16 h at 12°C. The fraction containing virions was diluted in DMEM and dialyzed against PBS. Purified virions were subjected to quantitative real-time PCR following dsRNA purification and the plaque-forming assay.

### Quantitative real-time PCR

Viral RNA was purified from whole-cell lysates or purified virions using a QIAamp Viral RNA Mini Kit. Viral cDNA was synthesized from positive-stranded RNA using RevertAid Reverse Transcriptase (Thermo Fisher Scientific) and a primer specific to the L1 gene positive-sense genome nucleotide position 1181–1204 (5’-ACTGAGGTTGCCAACGAACGGATG-3’). Viral RNA copy numbers were quantified using TaqMan Fast Universal PCR Master Mix (2×), no AmpErase UNG (Thermo Fisher Scientific), and the ViiA7 real-time PCR system (Life Technologies). The following primers and probes specific to the PRV L1 gene segment were used: forward primer, 5’-ACGCATCCTTCTGTGGGTC-3’; reverse primer, 5’-GAGATGGAGATGAAAGGTGTGAGTG-3’; probe, 5’-FAM-CCAAAGCTATAACAGTACCGTCTC-TAMRA-3’ (Eurofins Genomics, Ebersberg, Germany). The T7-MB L1 plasmid was used to generate a standard curve.

### mRNA translation assay

mRNA of NanoLuc gene was synthesized from pT7-secNLuc using mMESSAGE mMACHINE T7 Ultra (Ambion, Thermo Fisher Scientific) according to the manufacturer’s instructions. Poly-A tailing was performed on synthesized mRNA, and products were purified by standard phenol–chloroform extraction. To examine the effect of FAST protein on mRNA translation, monolayers of 293T cells in 24-well plates were transfected with 0.5 μg of pCAG-FAST-p10 or empty pCAGGS plasmid with 2 μl of TransIT-LT1 transfection reagent per 1 μg of plasmid DNA. At 2 h post transfection, culture medium was replaced with DMEM containing 5% FBS, and cells were transfected with 100 ng of NLuc mRNA using 2 μl of TransIT-mRNA (Mirus) per 1 μg of RNA. At 8, 12, 24, and 36 h post transfection of NLuc mRNA, NLuc activity in cell lysate was measured using Nano-Glo Luciferase Assay kit (Promega) and a luminometer (AB-2200, Atto, Tokyo, Japan) according to the manufacturers’ instructions.

### Cytotoxicity assay

Monolayers of Vero cells in 96-well plates were transfected with FAST-p10 expression vector or empty vector (0.1 μg/well) and incubated for various durations. At the indicated time points post transfection, cell viability was determined by the wst-1 assay. In brief, 2 μl of cell proliferation reagents was added to each well and samples were incubated at 37°C for 1 h. Optical density at 440 nm was measured using a microplate reader (Powerscan HT, DS Pharma Biomedical, Osaka, Japan).

### Animal experiments

Male 4-week-old C3H/HeNCrl (C3H) mice were purchased from Charles River Japan Inc. (Kanagawa, Japan). To obtain Kaplan–Meier survival curves, male C3H mice (n = 10 / group) were intranasally infected with 2.0 × 10^5^ PFU per animal of rsMB or rsMB-ΔFAST and observed for up to 18 days post infection. Bodyweight was recorded every 1–2 days. To assess viral replication and to generate survival curves in mice, 4-week-old male C3H mice (*n* = 5/group) were intranasally infected with 2.0 × 10^5^ PFU per animal of rsMB or rsMB-ΔFAST. Animals were euthanized 5 days after intranasal infection. Lungs were homogenized with a bead homogenizer (BeadSmash 12, Waken B Tech, Kyoto, Japan). Viral titers in lungs were determined by plaque assay. For histopathological study, C3H mice intranasally infected with 2 × 10^5^ PFU per animal of rsMB or rsMB-ΔFAST were euthanized at day 4 and lungs were fixed in 10% neutral buffered formalin solution and processed for pathological examination. Tissue sections were examined by hematoxylin and eosin staining and immunohistochemical analysis with rabbit anti-PRV-MB sigmaC primary antibody, horseradish peroxidase-conjugated secondary antibody, and diaminobenzidine tetrahydrochloride.

To evaluate the FAST mutant PRV as live attenuated vaccine, 3-week-old male C3H mice (n = 6/group) were intranasally infected with 4 × 10^5^ PFU of rsMB, rsMB-ΔFAST or rsMB-FAST (A30G) once or twice with a 1 week interval. Control mice were intranasally inoculated with PBS. At 14 days after the first infection (7 days after the second infection), mice were intranasally infected with 4 × 10^5^ PFU of rsMB. The survival rate and body weight were recorded for 2 weeks after challenge infection.

To obtain anti-PRV-MB serum, C3H mice that survived after intranasal infection of 2 × 10^5^ PFU of rsMB were intranasally re-infected two or four times with 2 × 10^6^ PFU of rsMB.

### Statistical analyses

Statistical analyses were performed using GraphPad Prism (v5.0, GraphPad). Viral replication was compared between two groups using the two-tailed Student’s *t*-test. The number of nuclei per syncytium was compared between two groups using the two-tailed Student’s *t*-test and between three or more groups using a one-way analysis of variance with Dunn’s or Dunnett’s multiple comparison test. Linear regression lines and R-square values were calculated for fusion efficiencies and viral titers of FAST-p10 mutant viruses. Survival curves were compared with that of the control group using the log rank test. Experiments were repeated at least three times. The sample numbers provided represent biological replicates. *p <* 0.05 was considered statistically significant.

### Ethics statement

The study was approved by the Animal Research Committee of Research Institute for Microbial Diseases, Osaka University (Approval number: Bi-Dou-25-04-0). The experiments were conducted following the guidelines for the Care and Use of Laboratory Animals of the Ministry of Education, Culture, Sports, Science and Technology, Japan. Chick-embryo fibroblasts prepared from 11-day-old chicken embryonated eggs were grown in DMEM supplemented with 10% chicken serum (Life Technologies, Thermo Fisher Scientific). The PRV-MB strain, which was isolated from an anonymous donor [52], was provided by the National Institute of Infectious Diseases (Tokyo, Japan) via a material transfer agreement. The usage of PRV-MB was approved by the Research Institute for Microbial Diseases, Osaka University (Approval number: 24-Biken-362).

## Supporting information

S1 Fig Generation of FAST-ORF-deletion mutant pteropine orthoreovirus (PRV). (*A*) Construction of FAST-ORF-deletion S1 gene segment. PRV strain MB S1-ΔFAST181 gene segment was generated by deleting 181 nucleotides (85–265) of the ORF encoding FAST-p10 protein. (*B*) Electrophoretic pattern of wild-type PRV (rsMB) and rsMB-ΔFAST181. Viral genomic double-stranded RNA was separated in an 8% polyacrylamide gel. Gene segments are indicated. (*C*) Monolayers of Vero cells were infected with rsMB or rsMB-ΔFAST181 and incubated for 12 h. Viral sigmaC protein was detected by indirect immunofluorescence staining. Scale bars are 100 μm.
(TIF)

S2 Fig Syncytium formation by pteropine orthoreovirus (PRV) FAST-p10 affects viral replication, cell morphology, cytotoxicity, and mRNA translation. (*A*) Replication of recombinant PRV in human cancer cell lines. Monolayers of cells were infected with wild-type (rsMB) or FAST-p10-deficient (rsMB-ΔFAST) PRV at a multiplicity-of-infection (MOI) of 0.001 plaque-forming units (PFU)/cell. At 72 h post infection, infectious virus titers in the cell lysates were determined. Data are expressed as means ± SD (*n* = 3) and were statistically analyzed using the *t*-test. (*B*) Time course of syncytium formation in Vero cells infected with rsMB. Green and red fluorescent Vero cells were prepared independently by transfection of plasmid expression vectors for enhanced green fluorescent protein (EGFP) or tdTomato. The cells were harvested by trypsinization, mixed, and spread. After settlement of cells, they were infected with rsMB at a MOI of 0.1 PFU/cell. Cells were fixed at indicated times post infection and observed by confocal microscopy. Representative data at 4, 5, and 6 h post infection are shown. Arrows indicate syncytia, which are positive for both red and green fluorescence. Scale bars are 100 μm. (*C*) Time course of rsMB replication and release of infectious virions. Vero cells were infected with rsMB at a MOI of 0.001 PFU/cell. At indicated times post infection, culture supernatant and cells were collected separately, and infectious virus titers were determined by plaque assay. Data are expressed as means ± SD (*n* = 3). (*D*) Cytotoxicity associated with FAST-p10 expression. Monolayers of Vero cells were transfected with FAST-p10 expression vector or empty vector. Cell viability was determined by wst-1 assay. Data are expressed as means ± SD (*n* = 3). * indicates *p* < 0.05 (*t*-test). (*E*) Monolayers of Vero cells in 48-well plates were transfected with 0.25 μg/well FAST-p10 expression vector or empty vector. After incubation for 2 h, cells were transfected with *in vitro*-synthesized single-stranded RNA encoding NanoLuc luciferase (NLuc). At 3, 8, 12, 24, and 36 h post transfection, NLuc activity in cell lysates was determined. NLuc values 36 h post transfection were statistically analyzed. Data are expressed as means ± SD (*n* = 3). NLuc activity at 36 h post transfection was statistically compared between the mock and FAST-p10 transfection groups using the *t*-test.
(TIF)

S3 Fig Cell–cell fusion activity correlates with viral replication. (*A*) Syncytium formation by pteropine orthoreovirus (PRV) mutants with different mutations affecting the FAST-p10 protein. Vero cells were infected with wild-type PRV (rsMB) or rsMB-FAST-p10 mutant strains. At 16 h post infection, cells were fixed and virus-infected foci were detected by immunostaining for viral sigmaC protein. Results for representative mutant viruses are shown. Arrows indicate virus-infected foci. Scale bars are 100 μm. (*B*) Positive correlation was observed between cell–cell fusion activities and virus propagation in HEK 293T cells. The cells were infected with FAST-p10 mutant viruses. Cell–cell fusion activities were determined by the number of nuclei involved in syncytia at 16 h post infection, and infectious virus titers were measured at 72 h post infection. Mean values of fusion activity and viral replication of the 25 strains of rsMB-FAST-p10 mutant viruses described in Fig 3 are plotted. A linear regression line and R-square value are shown. (*C*) Inhibition of cell–cell fusion by lysophosphatidylcholine (LPC). Vero cells were infected with rsMB at a MOI of 0.01 PFU/cell. At 2 h post infection, medium was changed to medium containing LPC (0–100 μM). Cells were fixed at 16 h post infection, and viral sigmaC protein was detected by immunostaining. Arrowheads indicate virus-infected foci without syncytium formation. Scale bars are 100 μm. (*D–G*) Replication kinetics of PRV in a cell–cell fusion-resistant cell line. (*D*) Lymphoid cell-like morphology of S180-meiji cells. Scale bar are 50 μm. (*E*) Morphological characterization of S180-Meiji cells after infection of rsMB and rsMB-ΔFAST. S180-Meiji cells were infected with rsMB and rsMB-ΔFAST at a MOI of 0.01 PFU/cell. At 24 h post infection, cells were fixed and viral antigens were detected by immunostaining with rabbit anti-sigmaC serum followed by anti-rabbit IgG-CF488 conjugates. Nuclei were stained with DAPI. Scale bars are 50 μm. (*F*) S180-Meiji cells were infected with rsMB or rsMB-ΔFAST at a MOI of 0.1 PFU/cell. At 0, 24, 48, and 72 h post infection, virus infectious titers in cell lysates were determined by the plaque assay. (*G*) S180-Meiji cells were infected with rsMB or rsMB-ΔFAST at a MOI of 0.1 PFU/cell. At 0, 6, 12, and 24 h post infection, viral antigens in whole-cell extracts were detected with an anti-sigmaA antibody. An anti-β-actin antibody was used as a loading control.
(TIF)

S4 Fig Syncytium formation promotes replication of FAST protein-deficient pteropine orthoreovirus (PRV). (*A*) Vero cells mock-transfected or transfected with PRV FAST-p10 expression vector were infected with FAST-p10-protein-deficient PRV (rsMB-ΔFAST) or wild-type PRV (rsMB). At 16 h post infection, cells were fixed and viral antigen (green) and plasma membrane (red) were visualized with antibodies to sigmaC and pan-cadherin, followed by Alexa488-conjugated or Alexa594-conjugated secondary antibodies, respectively. Arrowheads indicate viral-antigen-positive single cells; arrows indicate viral-antigen-positive syncytia. Cell nuclei were stained with 4',6-diamidino-2-phenylindole (DAPI). Scale bars are 20 μm. (*B*) Enhancement of viral replication by recombinant PRV FAST-p10 protein. Vero cells were transfected with the PRV FAST-p10 expression vector or empty vector (1.0 μg/well) at 2 h before infection with rsMB-ΔFAST at a MOI of 0.01, 0.1, 1, or 10 PFU/cell. Infectious virus titers in cell lysates at 16 h post infection were determined. Data are expressed as means ± SD (n = 4) and were statistically analyzed using the *t*-test. (*C*) Enhancement of viral replication by recombinant ARV FAST-p10 protein. Vero cells were transfected with the ARV FAST-p10 expression vector or empty vector (1.0–4.0 μg/well) at 2 h before infection with rsMB-ΔFAST at a MOI of 0.001 PFU/cell. (*D*) Vero cells were transfected with the ARV FAST-p10 expression vector or empty vector (1.0/well) at the indicated times points before infection with rsMB-ΔFAST at a MOI of 0.001 PFU/cell. Infectious virus titers in cell lysates at 16 h post infection were determined. Data are expressed as means ± SD (n = 3) and were statistically analyzed using the *t*-test. (*E*, *F*) Syncytium formation. BSR cells were transfected with expression vectors for (*E*) PRV FAST-p10 or (*F*) Sendai virus (SeV) modified recombinant F (Fc) and HN protein. At 12 and 24 h post transfection, cells were fixed in 3.8% formaldehyde and observed under a phase contrast microscope. Syncytia are indicated by arrowheads. Scale bars are 100 μm. (*G*) BSR cells were mock-transfected or transfected with FAST-p10 or SeV Fc and HN expression plasmids, and then infected with rsMB-ΔFAST or rsMB. At 16 h post infection, cells were fixed and viral antigen (green) was visualized with anti-rsMB sigmaC antibody followed by CF488-conjugated secondary antibody. Cell nuclei were stained with DAPI. Arrowhead indicates syncytium without viral antigen. Scale bars are 100 μm. (*H–J*) Effect of p17 and sigmaC overexpression on PRV FAST-p10 replication. Vero cells were transfected with the (*H*) p17 or (*I*) sigmaC expression vector or empty vector (1.0 μg/well) at 2 h before infection with a FAST-p10-deficient PRV mutant (rsMB-ΔFAST) at a MOI of 0.01 PFU/cell. Data are expressed as means ± SD (n = 4) and were statistically analyzed using the *t*-test. (*J*) Expression of viral proteins in cell lysates at 16 h post infection was determined by western blotting.
(TIF)

S5 Fig Structure and amino acid sequences of FAST proteins encoded in the genus *Orthoreovirus*. (*A*) Topological organization model of FAST proteins of pteropine orthoreovirus (PRV), avian orthoreovirus (ARV), reptilian orthoreovirus (RRV), and baboon orthoreovirus (BRV) (2). (*B*) Alignment of deduced amino acid sequences of FAST proteins. Functional motifs, as described elsewhere (2), are indicated. GenBank accession numbers used were as follows: PRV FAST-p10, AB521793; ARV FAST-p10, AAF45151; RRV FAST-p14, AY238887; BRV FAST-p15, AF406787.
(TIF)

S6 Fig Replication of a rotavirus in cells supportive or resistant to replication. MA104 or Vero cells were infected with group A rotavirus (RVA) strain SA11 at a multiplicity-of-infection of 0.01 plaque-forming units (PFU)/cell. After adsorption at 37°C for 1 h, cells were washed twice and incubated in Dulbecco’s modified Eagle’s medium supplemented with 0–1.0 μg/ml trypsin. At the indicated times post infection, cells were disrupted by repeated freeze–thaw cycles. Infectious virus titers were determined by plaque-formation assay. Data are expressed as means ± SD (*n* = 3).
(TIF)

S7 Fig Use of FAST-p10-deficient virus as live attenuated vaccine. (*A*) C3H mice (male, 3-week-old, *n* = 6/group) were intranasally infected with 4 × 10^5^ plaque-forming units (PFU) of pteropine orthoreovirus (PRV) with a mutation encoding a single amino acid substitution in the FAST-p10 protein (rsMB-FAST/A30G) once (on day 1; ×1) or twice (on day 1 and day 7; ×2). Control mice were intranasally inoculated with phosphate-buffered saline. On day 14, mice were intranasally infected with a lethal dose of wild-type PRV (rsMB, 4 × 10^5^ PFU). Survival of animals was monitored daily for 14 days after challenge infection. Black arrows indicate immunization; white arrows indicate challenge infection. Survival curves were statistically compared with that in the control group using the log rank test. (*B*) Bodyweight changes were determined in animals in each experimental group. Data are expressed as mean ± SD (*n* = 6).
(TIF)
