# Supplementary figures and images for: Cell–cell fusion induced by reovirus FAST proteins enhances replication and pathogenicity of non-enveloped dsRNA viruses
Source: PLoS Pathog. 2019 Apr 25;15(4):e1007675. doi: 10.1371/journal.ppat.1007675 (PMC6504114; doi:10.1371/journal.ppat.1007675)

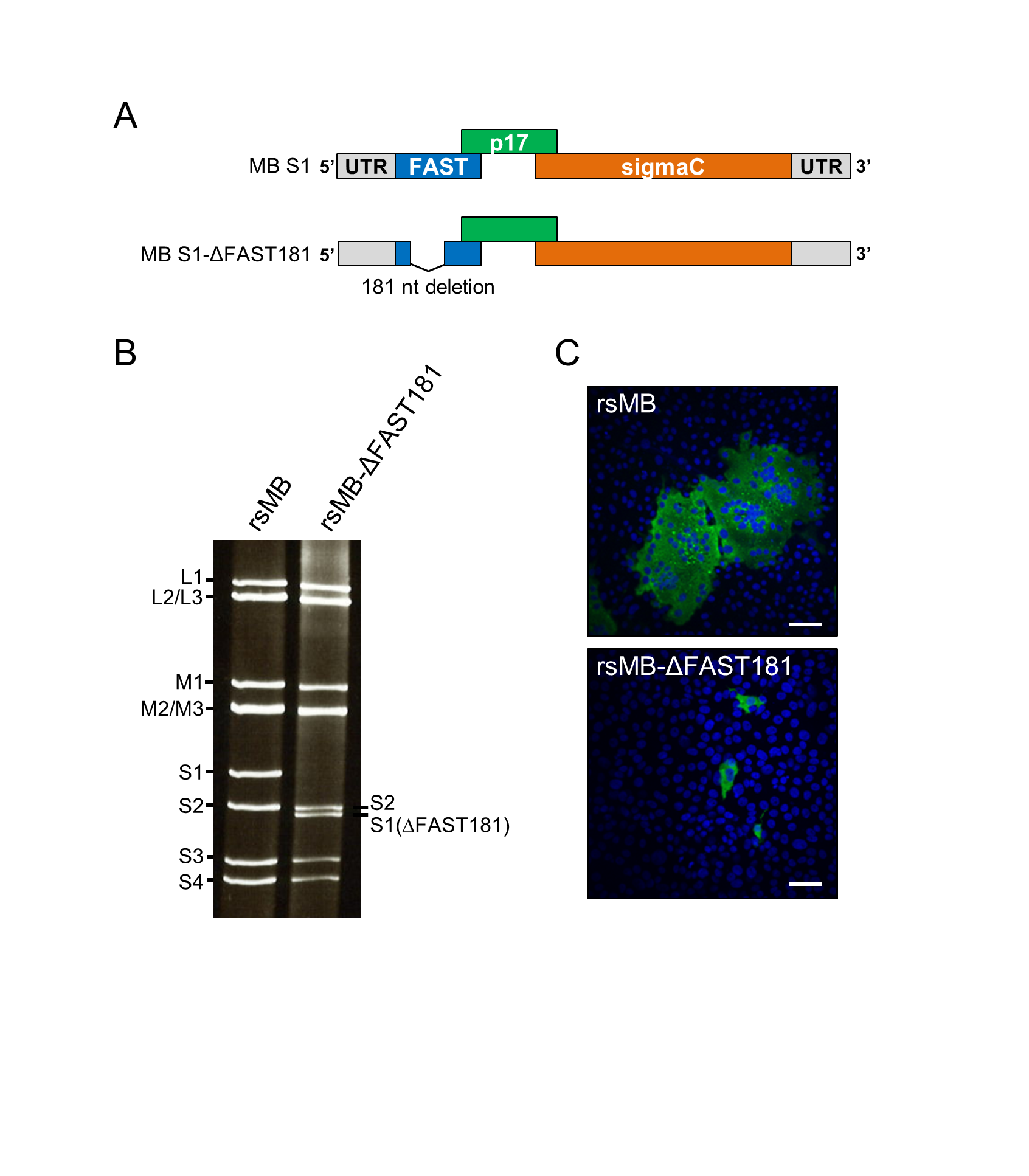

Supplement: S1 Fig — (A) Construction of FAST-ORF-deletion S1 gene segment. PRV strain MB S1-ΔFAST181 gene segment was generated by deleting 181 nucleotides (85–265) of the ORF encoding FAST-p10 protein. (B) Electrophoretic pattern of wild-type PRV (rsMB) and rsMB-ΔFAST181. Viral genomic double-stranded RNA was separated in an 8% polyacrylamide gel. Gene segments are indicated. (C) Monolayers of Vero cells were infected with rsMB or rsMB-ΔFAST181 and incubated for 12 h. Viral sigmaC protein was detected by indirect immunofluorescence staining. Scale bars are 100 μm. (TIF) [file ppat.1007675.s001.TIF]

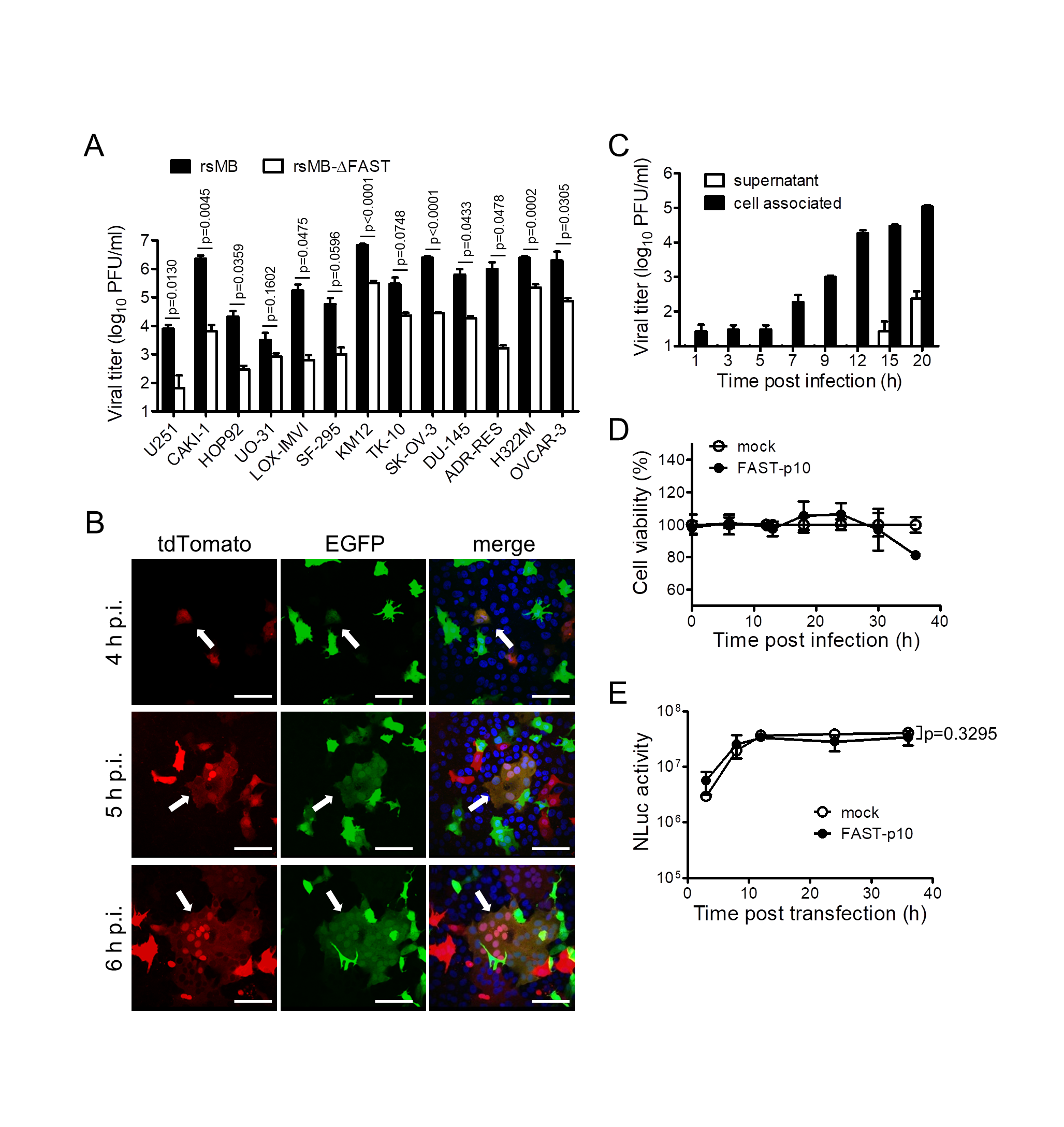

Supplement: S2 Fig — (A) Replication of recombinant PRV in human cancer cell lines. Monolayers of cells were infected with wild-type (rsMB) or FAST-p10-deficient (rsMB-ΔFAST) PRV at a multiplicity-of-infection (MOI) of 0.001 plaque-forming units (PFU)/cell. At 72 h post infection, infectious virus titers in the cell lysates were determined. Data are expressed as means ± SD (n = 3) and were statistically analyzed using the t-test. (B) Time course of syncytium formation in Vero cells infected with rsMB. Green and red fluorescent Vero cells were prepared independently by transfection of plasmid expression vectors for enhanced green fluorescent protein (EGFP) or tdTomato. The cells were harvested by trypsinization, mixed, and spread. After settlement of cells, they were infected with rsMB at a MOI of 0.1 PFU/cell. Cells were fixed at indicated times post infection and observed by confocal microscopy. Representative data at 4, 5, and 6 h post infection are shown. Arrows indicate syncytia, which are positive for both red and green fluorescence. Scale bars are 100 μm. (C) Time course of rsMB replication and release of infectious virions. Vero cells were infected with rsMB at a MOI of 0.001 PFU/cell. At indicated times post infection, culture supernatant and cells were collected separately, and infectious virus titers were determined by plaque assay. Data are expressed as means ± SD (n = 3). (D) Cytotoxicity associated with FAST-p10 expression. Monolayers of Vero cells were transfected with FAST-p10 expression vector or empty vector. Cell viability was determined by wst-1 assay. Data are expressed as means ± SD (n = 3). * indicates p < 0.05 (t-test). (E) Monolayers of Vero cells in 48-well plates were transfected with 0.25 μg/well FAST-p10 expression vector or empty vector. After incubation for 2 h, cells were transfected with in vitro-synthesized single-stranded RNA encoding NanoLuc luciferase (NLuc). At 3, 8, 12, 24, and 36 h post transfection, NLuc activity in cell lysates was determined. NL [file ppat.1007675.s002.TIF]

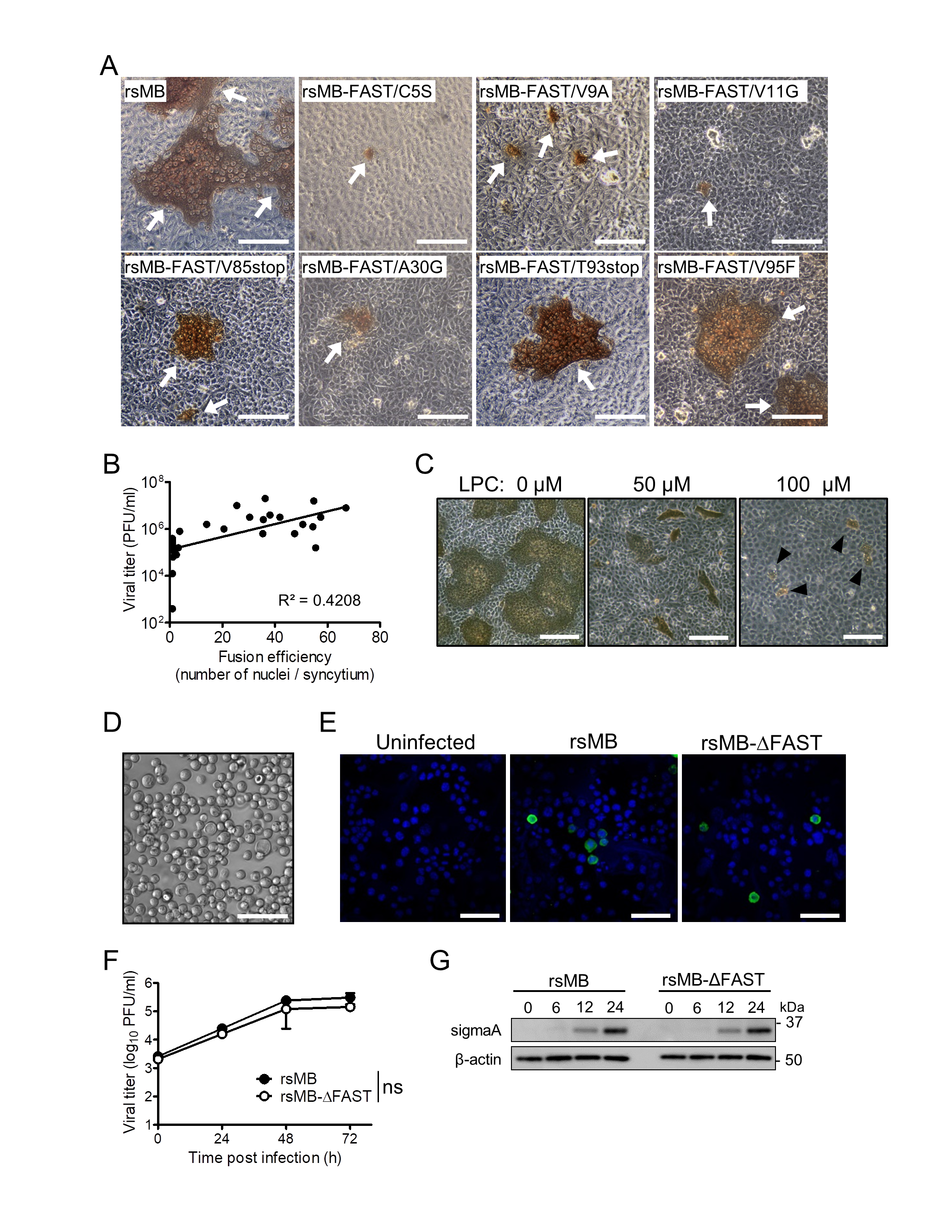

Supplement: S3 Fig — (A) Syncytium formation by pteropine orthoreovirus (PRV) mutants with different mutations affecting the FAST-p10 protein. Vero cells were infected with wild-type PRV (rsMB) or rsMB-FAST-p10 mutant strains. At 16 h post infection, cells were fixed and virus-infected foci were detected by immunostaining for viral sigmaC protein. Results for representative mutant viruses are shown. Arrows indicate virus-infected foci. Scale bars are 100 μm. (B) Positive correlation was observed between cell–cell fusion activities and virus propagation in HEK 293T cells. The cells were infected with FAST-p10 mutant viruses. Cell–cell fusion activities were determined by the number of nuclei involved in syncytia at 16 h post infection, and infectious virus titers were measured at 72 h post infection. Mean values of fusion activity and viral replication of the 25 strains of rsMB-FAST-p10 mutant viruses described in Fig 3 are plotted. A linear regression line and R-square value are shown. (C) Inhibition of cell–cell fusion by lysophosphatidylcholine (LPC). Vero cells were infected with rsMB at a MOI of 0.01 PFU/cell. At 2 h post infection, medium was changed to medium containing LPC (0–100 μM). Cells were fixed at 16 h post infection, and viral sigmaC protein was detected by immunostaining. Arrowheads indicate virus-infected foci without syncytium formation. Scale bars are 100 μm. (D–G) Replication kinetics of PRV in a cell–cell fusion-resistant cell line. (D) Lymphoid cell-like morphology of S180-meiji cells. Scale bar are 50 μm. (E) Morphological characterization of S180-Meiji cells after infection of rsMB and rsMB-ΔFAST. S180-Meiji cells were infected with rsMB and rsMB-ΔFAST at a MOI of 0.01 PFU/cell. At 24 h post infection, cells were fixed and viral antigens were detected by immunostaining with rabbit anti-sigmaC serum followed by anti-rabbit IgG-CF488 conjugates. Nuclei were stained with DAPI. Scale bars are 50 μm. (F) S180-Meiji cells were infected with rsMB or rsMB-ΔFAST at a MOI [file ppat.1007675.s003.TIF]

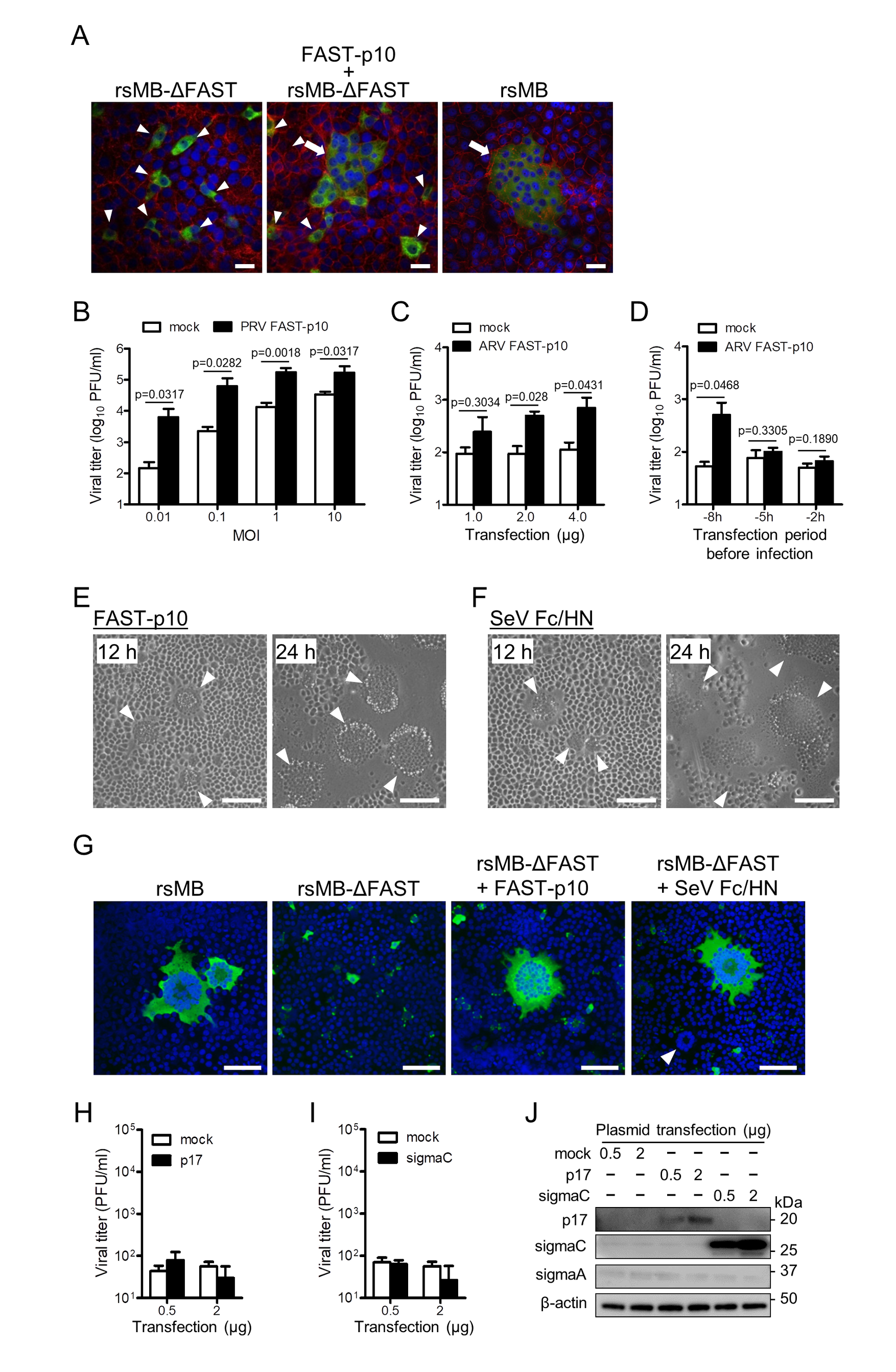

Supplement: S4 Fig — (A) Vero cells mock-transfected or transfected with PRV FAST-p10 expression vector were infected with FAST-p10-protein-deficient PRV (rsMB-ΔFAST) or wild-type PRV (rsMB). At 16 h post infection, cells were fixed and viral antigen (green) and plasma membrane (red) were visualized with antibodies to sigmaC and pan-cadherin, followed by Alexa488-conjugated or Alexa594-conjugated secondary antibodies, respectively. Arrowheads indicate viral-antigen-positive single cells; arrows indicate viral-antigen-positive syncytia. Cell nuclei were stained with 4',6-diamidino-2-phenylindole (DAPI). Scale bars are 20 μm. (B) Enhancement of viral replication by recombinant PRV FAST-p10 protein. Vero cells were transfected with the PRV FAST-p10 expression vector or empty vector (1.0 μg/well) at 2 h before infection with rsMB-ΔFAST at a MOI of 0.01, 0.1, 1, or 10 PFU/cell. Infectious virus titers in cell lysates at 16 h post infection were determined. Data are expressed as means ± SD (n = 4) and were statistically analyzed using the t-test. (C) Enhancement of viral replication by recombinant ARV FAST-p10 protein. Vero cells were transfected with the ARV FAST-p10 expression vector or empty vector (1.0–4.0 μg/well) at 2 h before infection with rsMB-ΔFAST at a MOI of 0.001 PFU/cell. (D) Vero cells were transfected with the ARV FAST-p10 expression vector or empty vector (1.0/well) at the indicated times points before infection with rsMB-ΔFAST at a MOI of 0.001 PFU/cell. Infectious virus titers in cell lysates at 16 h post infection were determined. Data are expressed as means ± SD (n = 3) and were statistically analyzed using the t-test. (E, F) Syncytium formation. BSR cells were transfected with expression vectors for (E) PRV FAST-p10 or (F) Sendai virus (SeV) modified recombinant F (Fc) and HN protein. At 12 and 24 h post transfection, cells were fixed in 3.8% formaldehyde and observed under a phase contrast microscope. Syncytia are indicated by arrowheads. Scale bars are 100 μm. (G) BSR [file ppat.1007675.s004.tif]

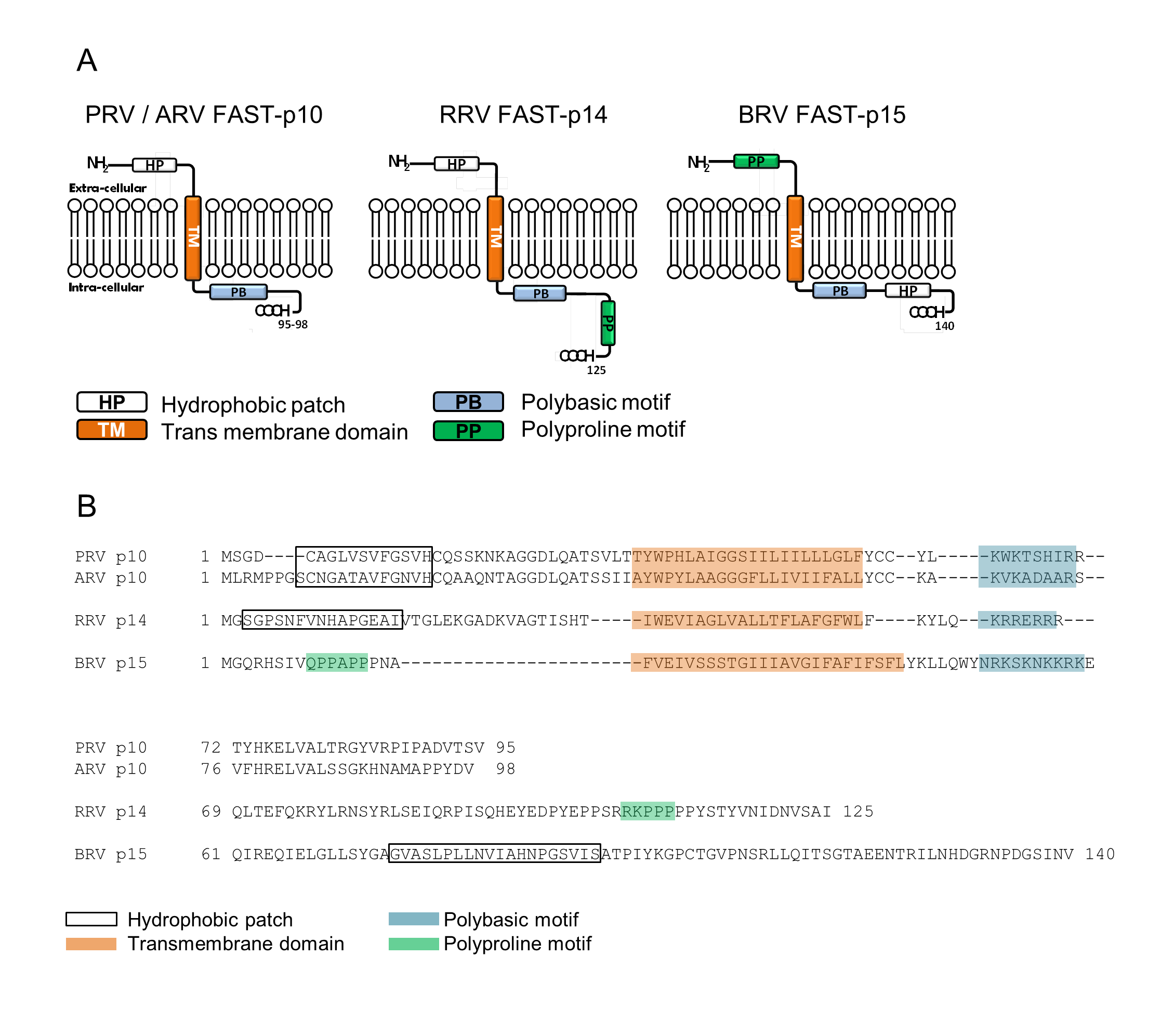

Supplement: S5 Fig — (A) Topological organization model of FAST proteins of pteropine orthoreovirus (PRV), avian orthoreovirus (ARV), reptilian orthoreovirus (RRV), and baboon orthoreovirus (BRV) (2). (B) Alignment of deduced amino acid sequences of FAST proteins. Functional motifs, as described elsewhere (2), are indicated. GenBank accession numbers used were as follows: PRV FAST-p10, AB521793; ARV FAST-p10, AAF45151; RRV FAST-p14, AY238887; BRV FAST-p15, AF406787. (TIF) [file ppat.1007675.s005.tif]

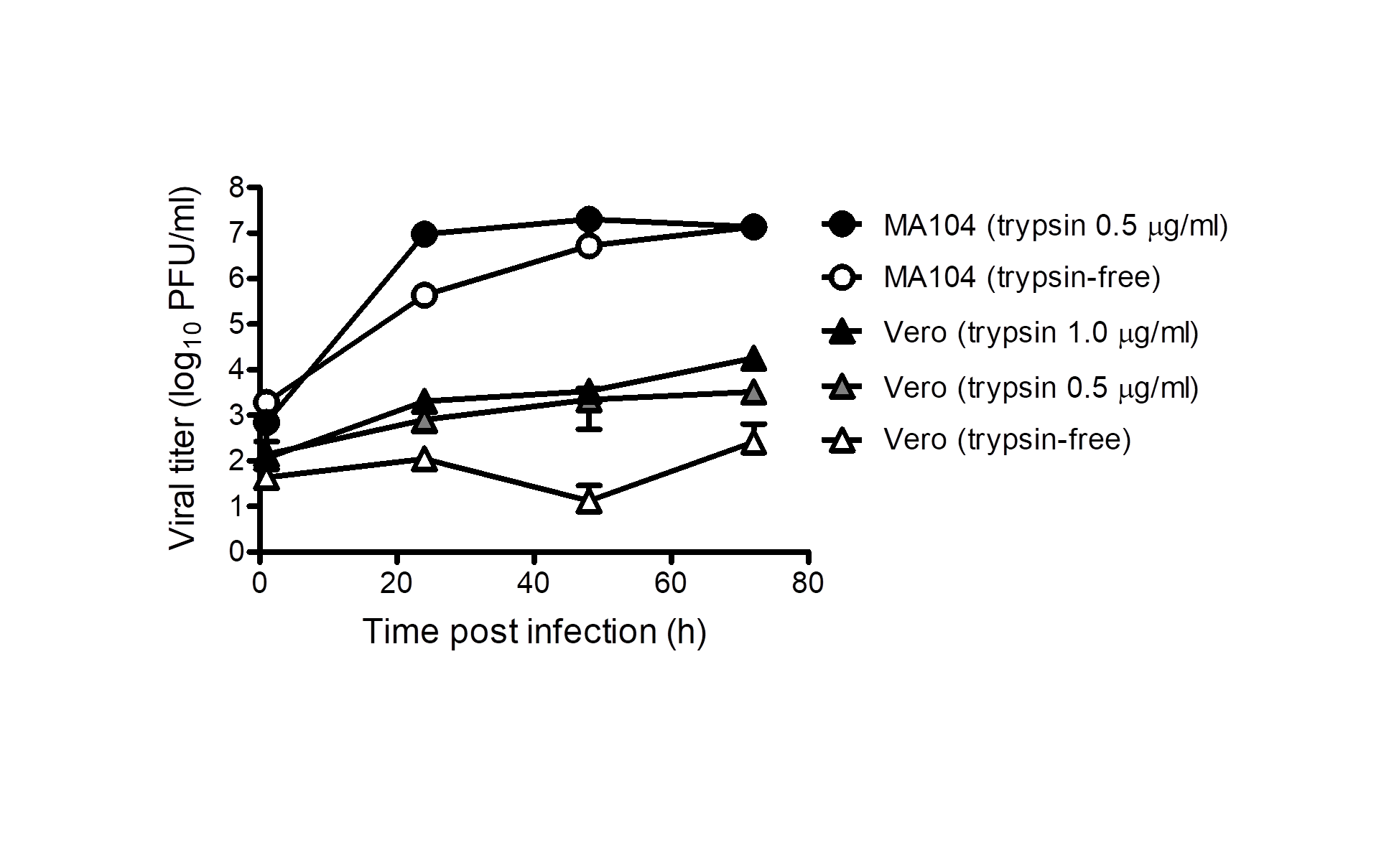

Supplement: S6 Fig — MA104 or Vero cells were infected with group A rotavirus (RVA) strain SA11 at a multiplicity-of-infection of 0.01 plaque-forming units (PFU)/cell. After adsorption at 37°C for 1 h, cells were washed twice and incubated in Dulbecco’s modified Eagle’s medium supplemented with 0–1.0 μg/ml trypsin. At the indicated times post infection, cells were disrupted by repeated freeze–thaw cycles. Infectious virus titers were determined by plaque-formation assay. Data are expressed as means ± SD (n = 3). (TIF) [file ppat.1007675.s006.TIF]

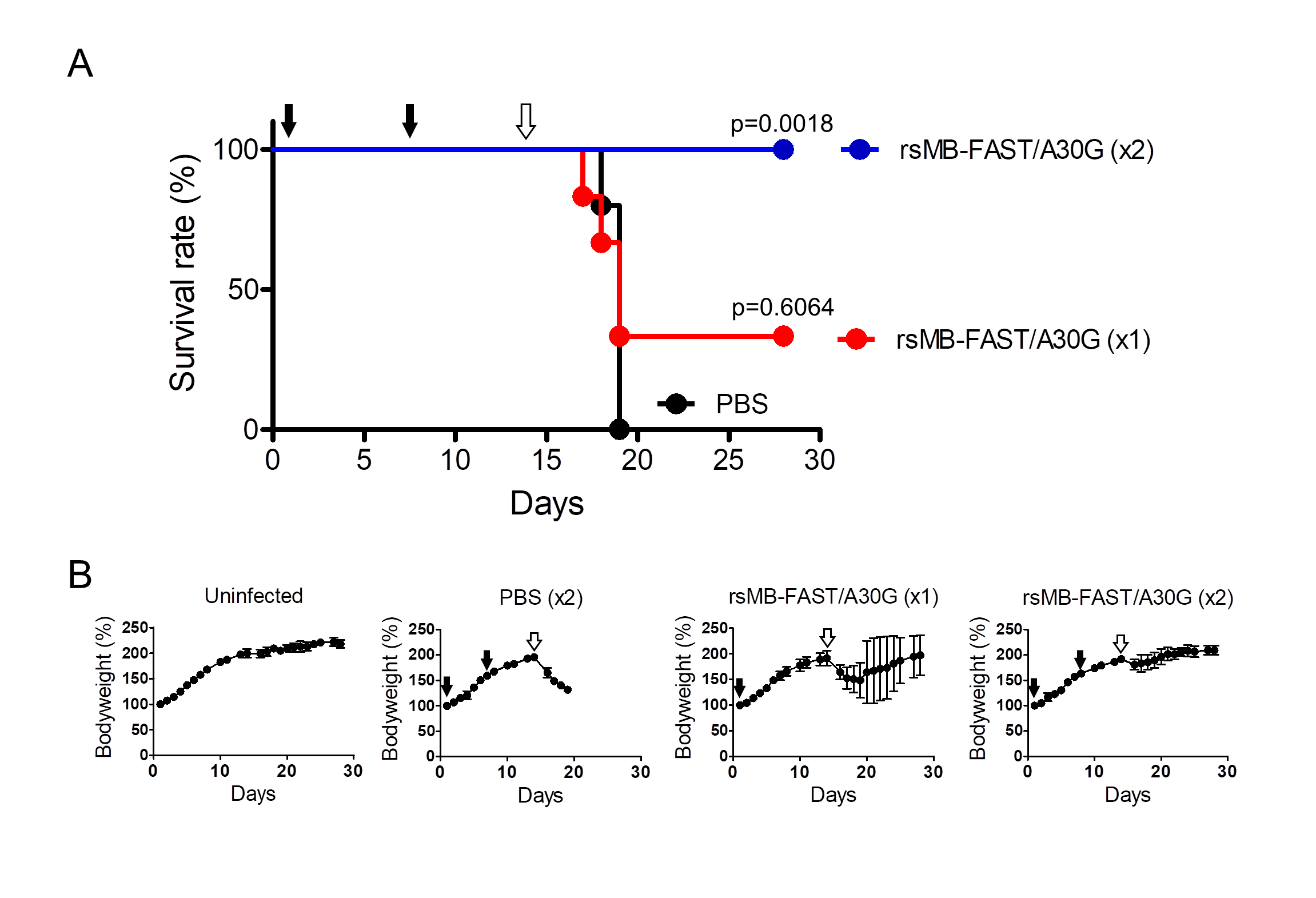

Supplement: S7 Fig — (A) C3H mice (male, 3-week-old, n = 6/group) were intranasally infected with 4 × 10^5^ plaque-forming units (PFU) of pteropine orthoreovirus (PRV) with a mutation encoding a single amino acid substitution in the FAST-p10 protein (rsMB-FAST/A30G) once (on day 1; ×1) or twice (on day 1 and day 7; ×2). Control mice were intranasally inoculated with phosphate-buffered saline. On day 14, mice were intranasally infected with a lethal dose of wild-type PRV (rsMB, 4 × 10^5^ PFU). Survival of animals was monitored daily for 14 days after challenge infection. Black arrows indicate immunization; white arrows indicate challenge infection. Survival curves were statistically compared with that in the control group using the log rank test. (B) Bodyweight changes were determined in animals in each experimental group. Data are expressed as mean ± SD (n = 6). (TIF) [file ppat.1007675.s007.TIF]
